# Peer Comparison or Guideline-Based Feedback and Postsurgery Opioid Prescriptions: A Randomized Clinical Trial

**DOI:** 10.1001/jamahealthforum.2024.0077

**Published:** 2024-03-15

**Authors:** Zachary Wagner, Allison Kirkegaard, Louis T. Mariano, Jason N. Doctor, Xiaowei Yan, Stephen D. Persell, Noah J. Goldstein, Craig R. Fox, Chad M. Brummett, Robert J. Romanelli, Kathryn Bouskill, Meghan Martinez, Kyle Zanocco, Daniella Meeker, Satish Mudiganti, Jennifer Waljee, Katherine E. Watkins

**Affiliations:** 1 RAND Corporation, Santa Monica, California; 2 RAND Corporation, Arlington, Virginia; 3 Sol Price School of Public Policy, University of Southern California, Los Angeles; 4 Palo Alto Medical Foundation, Palo Alto, California; 5 Division of General Internal Medicine, Department of Medicine, Center for Primary Care Innovation, Institute for Public Health and Medicine, Feinberg School of Medicine, Northwestern University, Chicago, Illinois; 6 Anderson School of Management, Department of Psychology, and Geffen School of Medicine, University of California at Los Angeles, Los Angeles; 7 University of Michigan Medical School, Ann Arbor; 8 RAND Europe, Westbrook Centre, Cambridge, United Kingdom; 9 Section of Endocrine Surgery, Division of General Surgery, Department of Surgery, David Geffen School of Medicine at UCLA, Los Angeles, California; 10 Keck School of Medicine, USC Leonard D. Schaeffer Center for Health Policy & Economics, Los Angeles, California; 11 Yale School of Medicine, New Haven, Connecticut

## Abstract

**Importance:**

Excess opioid prescribing after surgery can result in prolonged use and diversion. Email feedback based on social norms may reduce the number of pills prescribed.

**Objective:**

To assess the effectiveness of 2 social norm-based interventions on reducing guideline-discordant opioid prescribing after surgery.

**Design, Setting, and Participants:**

This cluster randomized clinical trial conducted at a large health care delivery system in northern California between October 2021 and October 2022 included general, obstetric/gynecologic, and orthopedic surgeons with patients aged 18 years or older discharged to home with an oral opioid prescription.

**Interventions:**

In 19 hospitals, 3 surgical specialties (general, orthopedic, and obstetric/gynecologic) were randomly assigned to a control group or 1 of 2 interventions. The guidelines intervention provided email feedback to surgeons on opioid prescribing relative to institutionally endorsed guidelines; the peer comparison intervention provided email feedback on opioid prescribing relative to that of peer surgeons. Emails were sent to surgeons with at least 2 guideline-discordant prescriptions in the previous month. The control group had no intervention.

**Main Outcome and Measures:**

The probability that a discharged patient was prescribed a quantity of opioids above the guideline for the respective procedure during the 12 intervention months.

**Results:**

There were 38 235 patients discharged from 640 surgeons during the 12-month intervention period. Control-group surgeons prescribed above guidelines 36.8% of the time during the intervention period compared with 27.5% and 25.4% among surgeons in the peer comparison and guidelines arms, respectively. In adjusted models, the peer comparison intervention reduced guideline-discordant prescribing by 5.8 percentage points (95% CI, −10.5 to −1.1; *P* = .03) and the guidelines intervention reduced it by 4.7 percentage points (95% CI, −9.4 to −0.1; *P* = .05). Effects were driven by surgeons who performed more surgeries and had more guideline-discordant prescribing at baseline. There was no significant difference between interventions.

**Conclusions and Relevance:**

In this cluster randomized clinical trial, email feedback based on either guidelines or peer comparison reduced opioid prescribing after surgery. Guideline-based feedback was as effective as peer comparison-based feedback. These interventions are simple, low-cost, and scalable, and may reduce downstream opioid misuse.

**Trial Registration:**

ClinicalTrials.gov NCT05070338

## Key Points

**Question:**

Can email feedback on opioid prescribing informed by social norms reduce guideline-discordant postoperative opioid prescribing?

**Findings:**

In this 3-arm cluster-randomized clinical trial that included 640 surgeons, 2 forms of social norm-based email feedback sent to surgeons with postoperative opioid prescriptions above guideline-recommended amounts significantly reduced guideline-discordant prescribing.

**Meaning:**

Email feedback based on social norms is an effective and light-touch intervention for increasing guideline-concordant opioid prescribing after surgery.

## Teaser

This cluster randomized clinical trial assesses the effectiveness of 2 social norm-based interventions on reducing guideline-discordant opioid prescribing after surgery.

## Introduction

Opioids prescribed after surgery are a significant driver of chronic opioid use and opioid use disorder.^1,2,3,4^ Postoperative opioid prescriptions also contribute to unused pills available for diversion to the community^5,6^; more than half of the opioids prescribed are never used by the patient and rarely disposed of safely.^7,8,9^ High-risk opioid prescribing is concentrated among surgeons who write the most prescriptions, with the top 5% of surgeons in terms of prescribing accounting for 40% of prescriptions written for large quantities.^10^ Despite widespread efforts in recent years to match opioid prescribing to patient need, excessive postoperative opioid prescribing has persisted.^8,9^

Providing surgeons with feedback on the quantity of opioids they prescribe relative to a social norm may help reduce opioid prescription quantities by challenging what is considered acceptable behavior.^11,12^ This could take the form of feedback that describes peer prescribing practices relative to an individual’s own (descriptive social norm) or feedback that provides information about opioid prescription quantities recommended by an authoritative source such as a guideline (injunctive social norm). Although previous randomized clinical trials have shown that social norms can be used to reduce inappropriate antibiotic prescribing among primary care clinicians^13^ and opioid prescribing in emergency departments,^14^ there is limited high-quality evidence on whether feedback based on social norms can influence opioid prescribing in the surgical setting, which has a different structure of care and has the second highest opioid prescribing rate of any specialty after pain medicine.^15,16^ Moreover, although recent research has examined the influence of either peer-based or guideline-based feedback (or both) on clinician behaviors,^17,18,19,20,21^ little is known regarding how these directly compare to one another.

To address these gaps, this study tests the effectiveness of 2 email feedback interventions relative to usual care across 3 surgical specialties in a multihospital cluster-randomized trial: (1) a descriptive social norm intervention that compares opioid prescription quantities of individual surgeons with that of their peers and (2) an injunctive social norm intervention that compares opioid prescription quantities to guideline recommendations.

## Methods

### Study Design and Oversight

This was a 3-arm cluster-randomized clinical trial conducted October 19, 2021, to October 18, 2022, in 3 surgical specialties (general, orthopedic, and obstetrics/gynecology) at 19 hospitals within Sutter Health, a large health care system in northern California. Details of the intervention and analysis plan were prespecified in a published protocol and are also available in Supplement 1.^22^ Activities were approved by the institutional review boards at Sutter Health and the RAND Corporation and monitored by an independent data safety and monitoring board. All data were obtained from the Sutter Health’s electronic health record (EHR) database. Gender and ethnic and racial categories were self-reported by patients; other racial category includes American Indian/Alaska Native, Native Hawaiian/Pacific Islander, other race, and multiple races. This study followed the Consolidated Standards of Reporting Trials (CONSORT) reporting guideline.

### Inclusion and Enrollment

All hospitals within the Sutter Health system as of May 2021 were enrolled in the study. To avoid spillover effects, facilities identified as regularly sharing surgical staff were grouped together, resulting in 19 hospitals for the analysis. Surgeons working exclusively in 1 of the 3 surgical specialties were eligible to participate in the study if they performed any surgical procedures for which opioid prescribing guidelines were available (guidelines described herein) at any of the 19 study hospitals between June 1, 2020, and May 31, 2021. Surgeons were included in the study if they also performed at least 1 surgical procedure for which opioid prescribing guidelines were available during the intervention period.

### Opioid Prescribing Guidelines

Both interventions as well as our primary outcome were based on prescribing guidelines developed by teams at the Mayo Clinic.^23,24,25,26^ These guidelines recommend procedure-specific ceilings for the total quantity of 5-mg oxycodone tablets needed over the postoperative recovery period (eTable 1 in Supplement 2). We converted these tablet quantities to morphine milligram equivalent (MME) quantities using the Centers for Disease Control and Prevention’s conversion table^27^ so that we could evaluate a prescription’s guideline compliance regardless of the type or strength of opioid prescribed.

### Intervention and Control Conditions

Both interventions provided feedback to surgeons if at least 2 of their patients were discharged with opioid prescriptions larger than the guideline-recommended amount in the prior month. Feedback was sent via emails from and signed by the surgeon’s department chair, chief medical executive, or chief of staff. Surgeons were not explicitly informed they were part of a study. We chose not to obtain informed consent from participants to avoid a Hawthorn effect and a waiver of informed consent was approved by the institutional review boards. However, hospital administrators could opt out of the intervention and surgeons could opt out of emails.

Although the content of the emails was distinct, the trigger for sending an email was identical: a surgeon having at least 2 patients with eligible discharge opioid prescriptions over guideline recommendations in the previous month. Eligible discharge prescriptions were those for adults discharged to home after a single surgical procedure with an applicable postoperative prescribing guideline, and where the prescription was for an oral medication. The email was sent to the operating surgeon even if the surgeon did not write the discharge prescriptions; 44% of discharge opioid prescriptions were written by someone other than the operating surgeon (usually a hospitalist) although the operating surgeon discharged the patient 84% of the time.

#### Peer Comparison (Descriptive Norm) Intervention

The subject line for the peer comparison email read “Your peers vs your opioid prescribing safety record.” The email content informed surgeons that their patients were prescribed opioid quantities exceeding the amounts prescribed by more than a particular percentage of their peers (eFigure 1 in Supplement 2). The peer percentage was calculated as the share of surgeons at Sutter Health in their specialty who had fewer than 2 patients discharged with opioid quantities exceeding guidelines in the past month, and ranged from 28% to 87% (mean, 61%) during the study period. The email also listed the procedures for which they prescribed higher quantities than their peers and the range prescribed by their peers for each procedure.

#### Guideline Feedback (Injunctive Norm) Intervention

The subject line of the guideline feedback email read “Best practice guidelines vs your opioid prescribing safety record.” The email content informed the surgeon that their patients were prescribed opioid quantities “exceeding the amounts recommended by safety guidelines for these procedures” (eFigure 2 in Supplement 2). The email also listed the procedures for which this occurred in the prior month and the guideline range for each procedure.

#### Control Condition

Surgeons in the control arm did not receive any emails and were not informed that they were being studied. We made this decision to avoid Hawthorn effects.

### Implementation

Each month, intervention emails were automatically created and sent to eligible surgeons via an email management platform. Sutter Health staff were responsible ensuring emails were sent out correctly.

### Randomization

Randomization occurred at the level of the surgical specialty. At each hospital, 1 specialty was randomized to each arm, stratified by hospital size and specialty. The allocation of specialties within hospitals to study arms was drawn using the sample function in R statistical software^28^ (version 4.3.2, R Foundation) applied to each block. We chose to randomize by specialty within hospital rather than at the hospital level to maximize the number of randomization clusters, which increases statistical power, and to avoid chance imbalance which is more likely with fewer randomization units. After randomization but before the start of the intervention, 1 hospital-specialty combination refused to participate in the peer comparison intervention owing to a hospital administrator’s concerns. To preserve the integrity of the randomized design, we treat the 43 discharges (<1% of sample) from this group as peer comparison although they did not receive the intervention.

### Outcome Measures

The primary prespecified outcome was the probability that a discharged patient was prescribed a quantity of opioids above the guideline for the respective procedure during the 12 intervention months. We extracted all initial discharge prescriptions from the EHR and defined a prescription as being above guidelines if the total MME quantity of opioids prescribed was above the ceiling of the guideline-recommended range. Surgeries where no opioids were prescribed were considered within guidelines.

We analyzed 2 prespecified secondary opioid prescribing outcomes: (1) the MME quantity prescribed at discharge, coded as zero when no opioid was prescribed and truncated at 500 MMEs (the 99th percentile), and (2) the probability that a discharged patient received any opioid prescription.

We also analyzed 3 prespecified outcomes that assess potential harmful effects of the interventions: (1) whether the patient got an additional opioid prescription filled in the 30 days after the surgery, (2) whether the patient had an emergency department visit in the 30 days after the surgery, and (3) whether the patient was hospitalized in the 30 days after the surgery.

### Statistical Analysis

We conducted 3 prespecified analyses.^22^ In each, we estimated intervention effects using the surgeon’s treatment assignment, regardless of whether the surgeon received an email during the study period. The first set of analyses modeled effects on primary and secondary outcomes at the level of the surgical specialty using a hierarchical linear model (HLM)^29^ with random effects for the surgeon and specialty within hospital and controlling for the baseline levels of the outcome (averaged by surgeon in the 24 months prior to the intervention). We included a version of the results controlling for a more comprehensive set of covariates in the eAppendix in Supplement 2. We used a logit-link for binary outcomes and reported absolute risk differences using recycled predictions.^30^ The second analysis estimated intervention effects over time by using separate HLMs for each of the 12 study months. The third set of analyses estimated effects for prespecified subgroups of surgeons based on 3 surgeon characteristics: (1) effects by surgical procedure volume during the intervention period, (2) effects by baseline guideline-discordant opioid prescribing in the 24 months before the intervention, and (3) effects by surgical specialty.

We also conducted 2 exploratory analyses. First, we estimated average effects at the level of the discharge using ordinary least squares (OLS) adjusted for the baseline level of the outcome and with standard errors clustered by randomization cluster.^31^ This discharge-level model weights each discharge equally and captures the average effect per discharged patient. Conversely, discharges from higher-volume surgeons tend to be downweighted in the HLM owing to their tendency toward higher variation in the outcome.^32^ Second, we used quantile regression to estimate the effect of the interventions on each decile of MMEs prescribed to determine whether findings were robust with a nonnormal distribution of MMEs prescribed.

The study was powered to detect a difference of 12 percentage points between each study arm and the control group. Baseline outcome measures were more strongly correlated with the outcome than the original conservative estimate, yielding additional power.^22^ All statistical analyses are described in more detail in the eAppendix in Supplement 2.

### Multiple Hypothesis Testing

We used false discovery rate (FDR) corrections to account for simultaneously tested hypotheses of the effect of both treatments.^33^ All reported *P* values have been adjusted for an FDR of .05.

## Results

**Figure 1.**
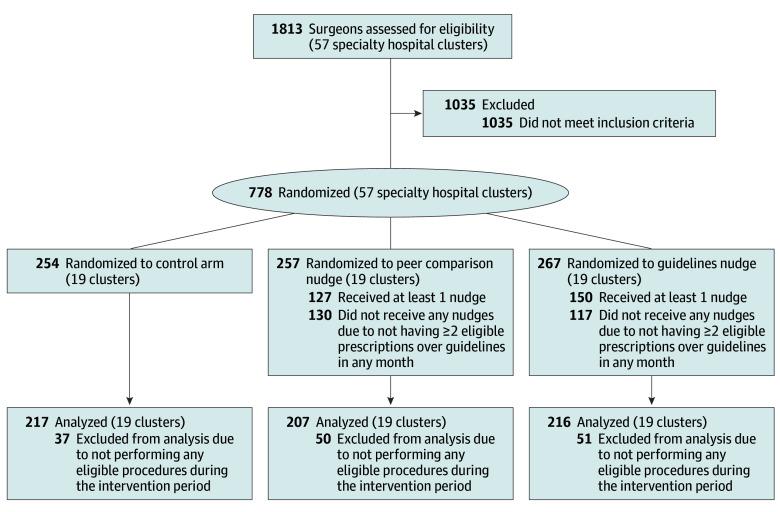
Consort Flow Diagram

Of the 778 surgeons eligible for inclusion in the study, 640 surgeons had 38 235 eligible discharges during the intervention period (Figure 1). Surgeon and patient characteristics were mostly balanced across arms (Table 1). The 3 arms were also balanced on the share of prescriptions above guideline quantities (primary outcome) and the share of discharges with any opioid prescribing in the 24 months leading up to the intervention. Although the guidelines arm had higher mean MMEs at baseline (100) relative to the other 2 arms (70 in control and 75 in peer comparison), median (IQR) MMEs were more balanced: 61 in guidelines (31-134), 57 in control (33-92), and 72 in peer comparison (35-99). More than 50% of total joint replacement procedures at baseline occurred in clusters assigned to guidelines, driving the guideline mean MME higher.

**Table 1.** Sample Characteristics^a^

Characteristic	No. (%)		
	Control	Peer comparison	Guidelines
Total hospitals, No.	19	19	19
Total surgeons, No.	217	207	216
Total patient discharges, No.	12 024	10 752	15 459
Surgeon characteristics			
General specialty	51 (23.5)	55 (27.0)	50 (23.1)
OB/GYN specialty	108 (49.7)	98 (47.3)	91 (42.1)
Orthopedic specialty	58 (26.7)	53 1 (25.6)	75 (34.7)
Years since receiving degree, mean (SD)	25.1 (10.9)	26.0 (10.6)	24.6 (11.9)
Gender			
Female	87 (40.0)	87 (42.0)	83 (38.4)
Male	122 (56.2)	109 (52.7)	128 (59.3)
Unknown	8 (3.7)	11 (5.3)	5 (2.3)
Patient characteristics			
Age, mean (SD), y	49.4 (17.6)	50.1 (17.3)	53.7 (17.7)
Medicaid beneficiary	1414 (11.7)	1475 (13.7)	2254 (14.5)
Length of stay, mean (SD), h	37.9 (58.1)	38.7 (53.5)	31.4 (56.4)
Body mass index, mean (SD)^b^	30.8 (7.93)	30.0 (7.32)	29.7 (6.95)
Pain condition	5948 (49.4)	5530 (51.4)	9159 (59.2)
Chronic opioid use	868 (7.21)	1219 (11.3)	1682 (10.8)
Opioid use past year	1873 (15.5)	1763 (16.3)	2853 (18.4)
Opioid use past month	1809 (15.0)	2439 (22.6)	3257 (21.0)
Opioid use past 24 h	3130 (26.0)	2808 (26.1)	4238 (27.4)
Gender^c^			
Female	9180 (76.3)	8383 (77.9)	10 577 (68.4)
Male	2839 (23.6)	2367 (22.0)	4878 (31.5)
Nonbinary	2 (0.016)	1 (0.009)	4 (0.025)
Race and ethnicity^c^			
Asian	1451 (12.0)	1527 (14.2)	1631 (10.5)
Black	949 (7.89)	450 (4.19)	764 (4.94)
White	6642 (55.2)	6554 (60.9)	9703 (62.7)
Other	2982 (24.8)	2220 (20.6)	3361 (21.7)
Outcomes at baseline (over 24 mo prior to intervention)			
Prescriptions above guidelines^c^	4491 (37.3)	4066 (37.8)	5516 (35.6)
MMEs, mean (SD)^d^	70.2 (59.2)	75.4 (51.9)	100.6 (97.1)
MMEs, median (range)	57.1 (0-338)	72.1 (0-500)	60.8 (0-447)
Any opioid prescription	6117 (50.8)	5512 (51.2)	8082 (52.2)

Abbreviations: MME, morphine milligram equivalent; OB/GYN, obstetrics and gynecology.

^a^ Data are from Sutter Health’s electronic health records.

^b^ Calculated as weight in kilograms divided by height in meters squared.

^c^ Gender and ethnic and racial categories were self-reported by patients; other racial category includes American Indian/Alaska Native, Native Hawaiian/Pacific Islander, other race, and multiple races.

^d^ The quantity of MMEs was coded as zero when no opioids were prescribed. Continuous variables show mean (SD).

### Intervention Effects on Above-Guideline Opioid Prescriptions

**Figure 2.**
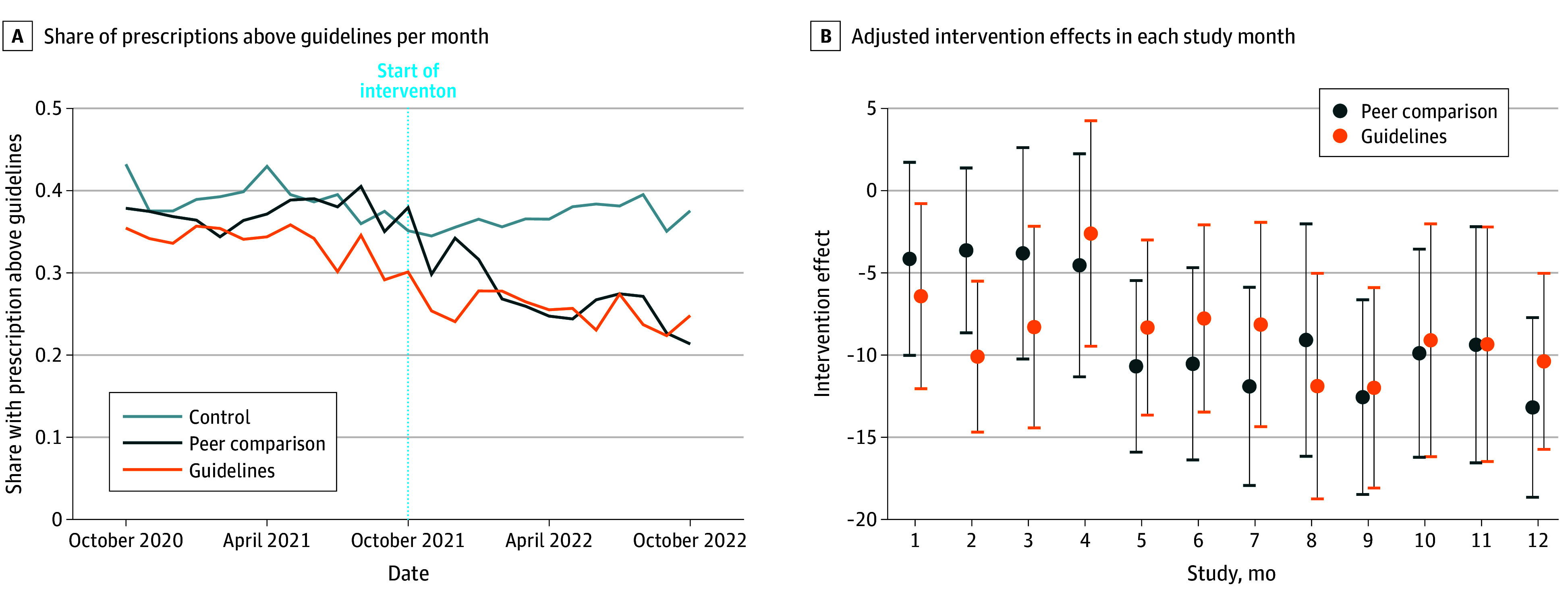
Prescribing Above Guideline Quantities Over Time A, The share of encounters with an opioid prescription at discharge exceeding guideline-recommended amounts. B, The effect of the intervention in each study month estimated using 12 separate hierarchical logistic models with random effects for surgeon and specialty within hospital. Intervention effects are absolute percentage point differences. Regression results for panel B are reported in eTable 2 in Supplement 2.

In the 12 months prior to the intervention, the share of discharges with an opioid prescription above guideline quantities was around 35% to 40% in all 3 arms (Figure 2A). During the intervention period, above guideline prescribing was 27.5% on average in the peer comparison arm and 25.4% in the guidelines arm compared with 36.8% in the control arm (Table 2). In adjusted hierarchical models (Table 2), the peer comparison intervention reduced the share of discharges with prescriptions above guideline quantities by 5.8 percentage points (95% CI, −10.5 to −1.1; *P* = .03) and the guidelines intervention reduced it by 4.7 percentage points (95% CI, −9.4 to −0.1; *P* = .05) compared with the control group. These constitute 17% and 14% relative reductions. Effect sizes were not significantly different between the 2 intervention arms. Figure 2B shows that effects became more pronounced over time in both arms, with 9 to 13 percentage point reductions in each of the past 4 months. Results were similar when regressions controlled for a more comprehensive set of covariates (eTable 3 in Supplement 2).

**Table 2.** Effect of Social Norms-Based Nudges on Primary and Secondary Outcomes^a^

Variable	Mean of outcome during 12 intervention months			Effect of peer comparison intervention, percentage points				Effect of guidelines intervention, percentage points			
	Control	Peer comparison	Guidelines	Unadjusted (95% CI)	*P* value	Adjusted (95% CI)	*P* value	Unadjusted (95% CI)	*P* value	Adjusted (95% CI)	*P* value
**Primary outcome**											
Opioid prescriptions above guideline quantities, %	36.8	27.5	25.4	−9.3 (−20.5 to 1.9)	.11	−5.8 (−10.5 to −1.1)	.03	−11.4 (−22.2 to −0.7)	0.08	−4.7 (−9.4 to −0.1)	.047
**Secondary outcomes**											
Morphine milligram equivalent, mean	67.7	58.4	78.7	−9.3 (−32.4 to 13.8)	.53	−4.6 (−18.8 to 9.6)	.94	10.9 (−23.0 to 44.8)	.53	−0.5 (−14.4 to 13.4)	.94
Any opioid prescribed, %	52.0	47.4	48.4	−4.6 (−16.7 to 7.5)	.55	0.4 (−4.1 to 4.8)	.90	−3.6 (−15.6 to 8.3)	.55	1.5 (−2.7 to 5.7)	.90

^a^ Unadjusted models compare means between each intervention arm and the control arm with standard errors clustered by specialty within hospital. Adjusted models include random effects for surgeon and specialty within hospital and control for baseline level of the outcome at the surgeon level. *P* values are adjusted for multiple testing using a false discovery rate of .05.

**Figure 3.**
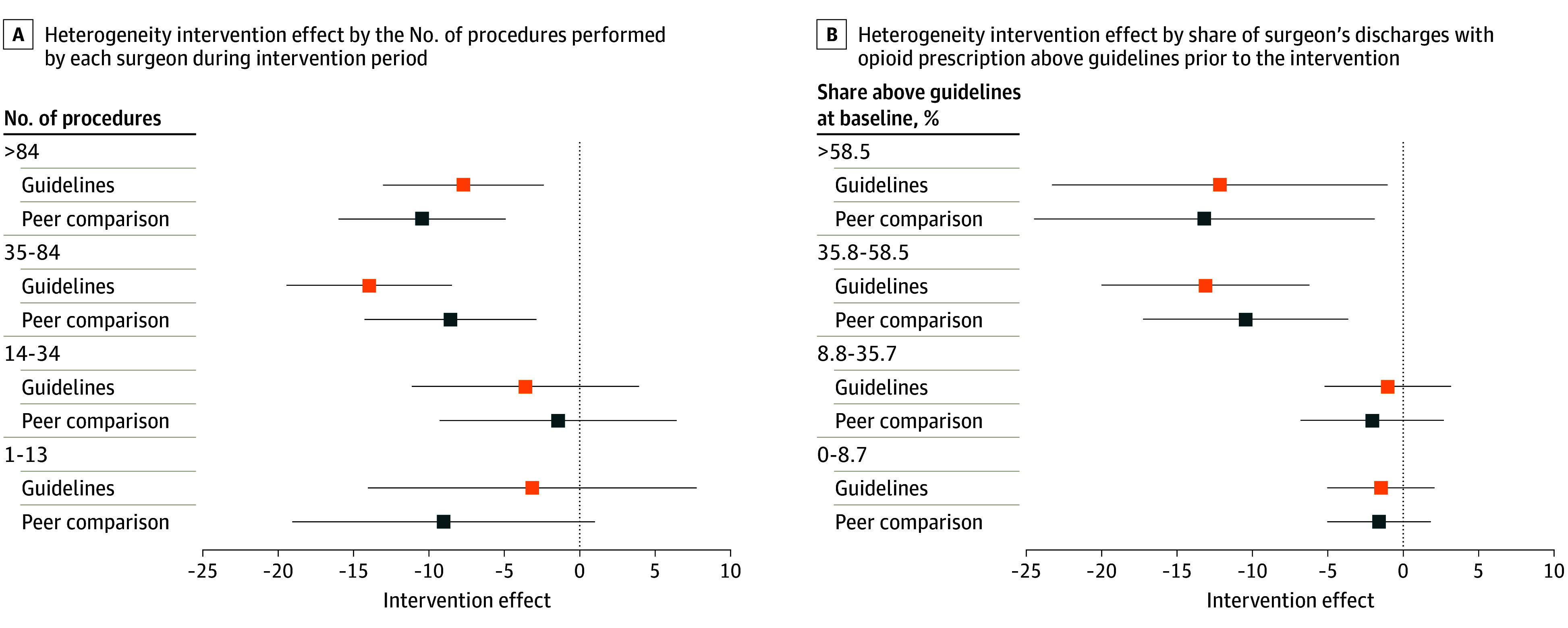
Heterogeneity in Intervention Effects by Procedure Volume and Baseline Guideline-Discordant Opioid Prescribing Intervention effects are shown in absolute percentage point differences. Each point estimate is the intervention effect from a separate hierarchal model including a logit link and random effects for surgeon and specialty within hospital and controlling for each surgeon’s share of discharges with guideline-discordant opioid prescriptions in the 24 months prior to the intervention. Subgroup categories are based on quartiles of each subgroup variable (described in more detail in the eAppendix in Supplement 2). Full regression results are reported in eTable 3 in Supplement 2. Effects sizes are significantly different between the top 2 and the bottom 2 categories for baseline prescribing but not for procedure volume (eTable 4 in Supplement 2). The whiskers indicate 95% CIs.

The effects among surgeons who performed above the median number of procedures during the intervention period were 8.8 and 9.5 percentage point reductions for peer comparison and guideline interventions, respectively (*P* < .001 for both; eTables 4 and 5 in Supplement 2; Figure 3), compared with 3.4 and 1.0 among surgeons with below-median numbers of procedures (*P* = .73 and *P* = .80, respectively; *P* values on difference in effect were .21 and .10, respectively). The effects among surgeons with above-median guideline-discordant prescribing at baseline were 10.6 and 10.9 percentage point reductions for peer comparison and guideline interventions, respectively (*P* = .005 for both), compared with 1.8 and 0.8 among surgeons with below-median guideline-discordant prescribing (*P* = .64 for both; *P* value on difference in effect was .03 for both). The effect was not significantly different across specialties (eTable 4 in Supplement 2).

In models analyzed at the discharge level, there was an absolute 9.7–percentage point reduction in guideline-discordant prescribing in the peer-comparison arm (95% CI, −14.5 to −4.9; *P* < .001) and a 10.1 percentage point reduction in the guidelines arm (95% CI, −15.6 to −4.7; *P* = .001) relative to the control group (eTable 6 in Supplement 2).

### Intervention Effects on MMEs Prescribed

During the intervention period, patients of surgeons in the control arm were prescribed 67.7 MMEs per discharge on average compared with 58.4 in the peer comparison arm and 78.7 in the guidelines arm (Table 2). Adjusted hierarchical models estimated nonsignificant reductions of 4.6 MMEs in the peer comparison arm (95% CI, −18.8 to 9.6; *P* = .94) and 0.5 MMEs in the guidelines arm (95% CI, −14.4 to 13.4; *P* = .94) compared with the control group. The discharge-level model estimated a 12.4 MME reduction in the peer comparison arm (95% CI, −24.2 to −0.6; *P* = .07) and a 12.2 MME reduction in the guidelines arm (95% CI, −25.2 to 0.80; *P* = .07; eTable 6 in Supplement 2). Quantile regressions estimated reductions at the median of 14.1 MMEs (95% CI, −21.0 to −7.3; *P* < .001) in the peer comparison arm and 13.8 in the guidelines arm (95% CI, −20.8 to −6.7; *P* < .001) compared with the control group, and reductions were larger at the higher end of the distribution (eFigure 3 in Supplement 2).

### Intervention Effects on the Probability of an Opioid Being Prescribed

Overall, 52% of discharges during the intervention period in the control group had any opioid prescription compared with 48% in both intervention arms. Adjusted hierarchical models estimated no significant differences between either intervention arm relative to the control group on the probability of prescribing any opioid (Table 2). The discharge-level model estimated a 4.9–percentage point reduction in any opioid prescribed at discharge in each intervention arm (peer comparison: 95% CI, −8.8 to −1.1; *P* = .02; guidelines: 95% CI, −9.0 to −0.8; *P* = .02; eTable 6 in Supplement 2).

### Intervention Effects on Patient Outcomes

We found no significant differences in the probability of getting an additional opioid prescription filled, having an emergency department visit, or being hospitalized in the 30 days after the discharge (eTable 7 in Supplement 2).

### Sensitivity Analyses

We conducted 2 sensitivity analyses. First, because only 56% of surgeons received an email during the study period, accounting for 81% of discharges (see eFigure 4 in Supplement 2), we conducted a sensitivity analysis where we restricted to surgeons who received an email at least once during the intervention period. Results were more pronounced for this subgroup (eTable 8 in Supplement 2). Second, we estimated the intervention effects restricting to only surgeons who wrote the opioid prescription, if an opioid was prescribed, or discharged the patient themselves, if an opioid was not prescribed. Effect sizes for this subgroup were similar to the main results (eTable 8 in Supplement 2).

## Discussion

### Principal Findings

Despite federal and state efforts, opioid overprescribing remains common in our setting, with 37% of postsurgical discharge prescriptions being above guideline quantities in our cohort before the intervention. Email feedback informing surgeons their patients were prescribed opioid amounts that exceeded institutionally endorsed guidelines or their peers’ prescriptions reduced guideline-discordant prescribing by about 5 percentage points relative to usual care. Effect sizes grew stronger over time (approximately a 10–percentage point reduction in the final 4 months of the intervention). These interventions were light-touch and incured minimal ongoing costs after implementation, yet were associated with clinically meaningful improvements in opioid prescribing. Both interventions could be scaled to any hospital system with an EHR that recorded opioid prescriptions at patient discharge.

### Implications for Public Health

A 5–percentage point reduction in guideline-discordant opioid prescribing could be important for public health outcomes. However, our prespecified analyses did not find significant effects on MMEs prescribed at discharge, which suggest the public health effects could be limited. In follow-up analysis, we found larger intervention effects (10–percentage point reductions) when we modeled effects at the discharge level (eTable 6 in Supplement 2) compared with the prespecified analyses, which included effects at the surgical specialty level. We also found significant reductions in MMEs prescribed and any opioids prescribed using the discharge-level model. This is because intervention effects were larger for surgeons with more discharges and discharges from higher-volume surgeons tend to be downweighted in the prespecified analyses.^34^ Moreover, high-volume surgeons contribute disproportionately to overprescribing.^10^ Thus, discharge-level analyses may be more relevant for assessing public health effects.

With 26 193 discharges in the intervention arms over the study year, estimates from the discharge-level model of a 12 MME reduction in opioids prescribed per discharged patient corresponded to 319 554 fewer MMEs or the equivalent of about 42 000 fewer pills of 5-mg oxycodone in Sutter Health patients’ communities over the study period.

### Implications for Scale-Up

Importantly, we found the guideline intervention was as successful as the peer comparison intervention. Going forward, guideline-based interventions to reduce excessive postoperative opioid prescribing may be preferable to peer-based interventions for several reasons. First, peer-comparison interventions are more challenging to implement because they require regularly updating feedback with current peer behavior. Second, although research suggests clinicians find peer comparison feedback helpful,^18^ some administrators in this study expressed concerns about peer comparison feedback. Third, guideline-based feedback can be implemented in smaller hospital systems where there are too few peers for a rich comparison.

### Comparison With Other Studies

To our knowledge, this is the first study to examine the effect of feedback interventions on postoperative opioid prescribing using a randomized design, and one of the first studies to compare the effectiveness of 2 types of social norms on clinician behavior—peer-comparison descriptive norms and guideline-based injunctive norms. Prior efforts to change postoperative prescribing with behavioral interventions have used pretest-posttest designs and have shown reductions in the number of tablets prescribed.^15,35,36,37,38,39^ The present work is consistent with trials testing clinician feedback interventions on opioid prescribing in emergency department, primary care, and urgent care sites,^14,17,40,41,42,43^ although 2 other trials examining clinician feedback on concurrent prescribing of opioids and benzodiazepines did not show reductions^40,44^ in concurrent prescribing. The finding that both the peer-based descriptive norm and guideline-based injunctive norm treatment conditions were equally effective contributes to an ongoing debate from the broader norms literature about which type of norm—descriptive or injunctive—is more likely to exert an effect in a fast-paced environment in which there is a great deal of cognitive depletion.^45,46^

### Limitations

This study has several limitations. First, we could not disentangle the effects of the social norms mechanisms from the effects of simply having one’s prescribing monitored. Of course, monitoring effects would also be present in any clinical version of the intervention (part of the intervention was informing surgeons that their opioid prescribing was being monitored), so this did not bias our estimates of overall intervention effects. Second, we did not assess patient outcomes such as self-reported pain levels, daily functioning, or quality of life. However, other work has not shown a relationship between reduced postoperative prescriptions and adverse patient outcomes,^39,47,48,49,50,51^ and patients in the intervention arms were not more likely to have an emergency department visit, an inpatient visit, or additional opioid prescription fill following surgery. Third, although we used a clustered design to avoid spillover, surgeons could talk across specialties within hospitals, which could have resulted in contamination and thus understated intervention effects. However, the lack of reduction in the control group suggests that the design prevented substantial spillover effects. Finally, the email was sent to the operating surgeon even when they did not make the discharge prescription. In some cases, surgeons might not have control over prescribing at discharge, and negative feedback for these surgeons could decrease receptivity to other institutional goals. This design choice was intended to avoid spillover (nonsurgeon prescribers are more likely to prescribe across specialties) and any scaled-up version of this intervention would likely send emails directly to the clinicians who make the discharge prescription.

## Conclusions

This 3-arm cluster-randomized clinical trial found that, among surgeons specializing in orthopedic, general, and obstetric/gynecological surgery, both email feedback informing surgeons that they ordered opioid prescriptions above guideline-recommended amounts and email feedback comparing their prescribing to that of their peers reduced guideline-discordant opioid prescribing compared with receiving no feedback.

## Notes

Supplement 1. Trial Protocol

Supplement 2. eAppendix eTable 1. Prescribing guidelines for procedures included in the present study eTable 2. Intervention impacts over time (full output from Figure 2B in main text) eTable 3. Intervention effects with full set of covariates included eTable 4. Subgroup analyses of intervention impacts on guideline-discordant prescribing eTable 5. Tests for differences in intervention impacts by subgroup eTable 6. Effect of social norms-based nudges on primary and secondary outcomes (discharge level) eTable 7. Intervention effects on patient outcomes eTable 8. Sensitivity analyses using surgeons who were more exposed to the intervention eFigure 1. Example of Peer Comparison Intervention Email eFigure 2. Example of Guidelines Intervention Email eFigure 3. Effect of social norms-based nudges on each quantile of MMEs prescribed at discharge eFigure 4. Exposure to interventions

Supplement 3. Data Sharing Statement
