# Supplementary material for: Peer Comparison or Guideline-Based Feedback and Postsurgery Opioid Prescriptions: A Randomized Clinical Trial
Source: JAMA Health Forum. 2024 Mar 15;5(3):e240077. doi: 10.1001/jamahealthforum.2024.0077 (PMC10943416; doi:10.1001/jamahealthforum.2024.0077)
Supplement: Supplement 1. — Trial Protocol [file jamahealthforum-e240077-s001.pdf]

Postoperative Nudges to Reduce Opioid Prescribing (POST-OP)

Protocol Number: R01DA046226

National Clinical Trial (NCT) Identified Number: NCT05070338

Principal Investigator: Katherine E. Watkins

Funded by: The National Institute on Drug Abuse

Version Number: v.1.1.1

12 October 2021

Summary of Changes from Previous Version:

**Affected Section(s)**	**Summary of Revisions Made**	**Rationale**
**6.1.1**	Updated intervention content and triggers	Intervention content and triggers were refined in light of feedback from Sutter Health hospital administrators and behavioral scientists on the study team

Table of Contents

STATEMENT OF COMPLIANCE 1
1 PROTOCOL SUMMARY 1
1.1 Synopsis 1
1.2 Schema 2
1.3 Schedule of Activities (SoA) 2
2 INTRODUCTION 3
2.1 Study Rationale 3
2.2 Background 3
2.3 Risk/Benefit Assessment 4
2.3.1 Known Potential Risks 4
2.3.2 Known Potential Benefits 4
2.3.3 Assessment of Potential Risks and Benefits 4
3 OBJECTIVES AND ENDPOINTS 4
4 STUDY DESIGN 5
4.1 Overall Design 5
4.2 Scientific Rationale for Study Design 5
4.3 Justification for Dose 5
4.4 End of Study Definition 6
5 STUDY POPULATION 6
5.1 Inclusion Criteria 6
5.2 Exclusion Criteria 6
5.3 Lifestyle Considerations 6
5.4 Screen Failures 6
5.5 Strategies for Recruitment and Retention 6
6 STUDY INTERVENTION 6
6.1 Study Intervention(s) Administration 6
6.1.1 Study Intervention Description 6
6.1.2 Dosing and Administration 8
6.2 Preparation/Handling/Storage/Accountability 8
6.2.1 Acquisition and accountability 8
6.2.2 Formulation, Appearance, Packaging, and Labeling 8
6.2.3 Product Storage and Stability 8
6.2.4 Preparation 8
6.3 Measures to Minimize Bias: Randomization and Blinding 9
6.4 Study Intervention Compliance 9
6.5 Concomitant Therapy 9
7 STUDY INTERVENTION DISCONTINUATION AND PARTICIPANT DISCONTINUATION/WITHDRAWAL 9
7.1 Discontinuation of Study Intervention 9
7.2 Participant Discontinuation/Withdrawal from the Study 9
7.3 Lost to Follow-Up 9
8 STUDY ASSESSMENTS AND PROCEDURES 10
8.1 Efficacy Assessments 10
8.2 Safety and Other Assessments 10
8.3 Adverse Events and Serious Adverse Events 11
8.3.1 Definition of Adverse Events (AE) 11

8.3.2 Definition of Serious Adverse Events (SAE) 11
8.3.3 Classification of an Adverse Event 12
8.3.4 Time Period and Frequency for Event Assessment and Follow-Up 12
8.3.5 Adverse Event Reporting 12
8.3.6 Serious Adverse Event Reporting 12
8.3.7 Reporting Events to Participants 13
8.3.8 Events of Special Interest 13
8.3.9 Reporting of Pregnancy 13
8.4 Unanticipated Problems 13
8.4.1 Definition of Unanticipated Problems (UP) 13
8.4.2 Unanticipated Problem Reporting 13
8.4.3 Reporting Unanticipated Problems to Participants 14
9 STATISTICAL CONSIDERATIONS 14
9.1 Statistical Hypotheses 14
9.2 Sample Size Determination 14
9.3 Populations for Analyses 14
9.4 Statistical Analyses 14
9.4.1 General Approach 14
9.4.2 Analysis of the Primary AND SECONDARY Efficacy EndpointS 15
9.4.3 Safety Analyses 16
9.4.4 Baseline Descriptive Statistics 16
9.4.5 Planned Interim Analyses 16
9.4.6 Sub-Group Analyses 17
9.4.7 Tabulation of Individual participant Data 17
9.4.8 Exploratory Analyses 17
10 SUPPORTING DOCUMENTATION AND OPERATIONAL CONSIDERATIONS 17
10.1 Regulatory, Ethical, and Study Oversight Considerations 17
10.1.1 Informed Consent 17
10.1.2 Study Discontinuation and Closure 17
10.1.3 Confidentiality and Privacy 18
10.1.4 Future Use of Stored Specimens and Data 18
10.1.5 Key Roles and Study Governance 18
10.1.6 Safety Oversight 18
10.1.7 Clinical Monitoring 18
10.1.8 Quality Assurance and Quality Control 18
10.1.9 Data Handling and Record Keeping 19
10.1.10 Protocol Deviations 19
10.1.11 Publication and Data Sharing Policy 20
10.1.12 Conflict of Interest Policy 20
10.2 Abbreviations 20
10.3 Protocol Amendment History 21
11 REFERENCES 22

STATEMENT OF COMPLIANCE

The trial will be carried out in accordance with International Conference on Harmonisation Good Clinical Practice (ICH GCP) and the following:

United States (US) Code of Federal Regulations (CFR) applicable to clinical studies (45 CFR Part 46, 21 CFR Part 50, 21 CFR Part 56, 21 CFR Part 312, and/or 21 CFR Part 812)

National Institutes of Health (NIH)-funded investigators and clinical trial site staff who are responsible for the conduct, management, or oversight of NIH-funded clinical trials have completed Human Subjects Protection and ICH GCP Training.

The protocol, informed consent form(s), recruitment materials, and all participant materials will be submitted to the Institutional Review Board (IRB) for review and approval. Approval of both the protocol and the consent form must be obtained before any participant is enrolled. Any amendment to the protocol will require review and approval by the IRB before the changes are implemented to the study. In addition, all changes to the consent form will be IRB-approved; a determination will be made regarding whether a new consent needs to be obtained from participants who provided consent, using a previously approved consent form.

1 PROTOCOL SUMMARY

1.1 SYNOPSIS

Title: Postoperative Nudges to Reduce Opioid Prescribing (POST-OP)

Study Description: This study tests the effectiveness of two email-based behavioral nudges, one based on peer behavior and one based on best practice guidelines, in reducing excessive opioid prescriptions after surgery. It will be conducted in three surgical specialties (general surgery, orthopedic surgery, and obstetric/gynecological surgery) at 19 hospitals within one healthcare system. At each hospital, these specialties will each be randomized to a control group or one of two nudge groups. Both types of email-based nudges are expected to reduce opioid prescribing after surgery.

Objectives: Primary Objective: Health Services Research
Secondary Objectives: Basic Science

Endpoints: Primary Endpoint: Share of discharge opioid prescriptions above prescribing guidelines
Secondary Endpoints:

- Morphine milligram equivalents (MMEs) prescribed at discharge
- Days' supply of opioids prescribed at discharge
- Share of discharges where any opioid was prescribed
- Share of patients on opioids for greater than 3 months post-discharge
- Number of 30-day all-cause emergency department visits
- Number of 30-day all-cause hospitalizations
- Share of discharge opioid prescriptions above prescribing guidelines in the year after the intervention ends

**Study Population:**	Surgeons in northern California
**Phase:**	N/A
**Description of Sites/Facilities Enrolling Participants:**	19 Sutter Health hospitals
**Description of Study Intervention:**	Each month for one year, surgeons in the nudge groups will receive emails comparing their opioid prescribing either to their peers' prescribing or to prescribing guidelines.
**Study Duration:**	24 months
**Participant Duration:**	12 months

1.2 SCHEMA

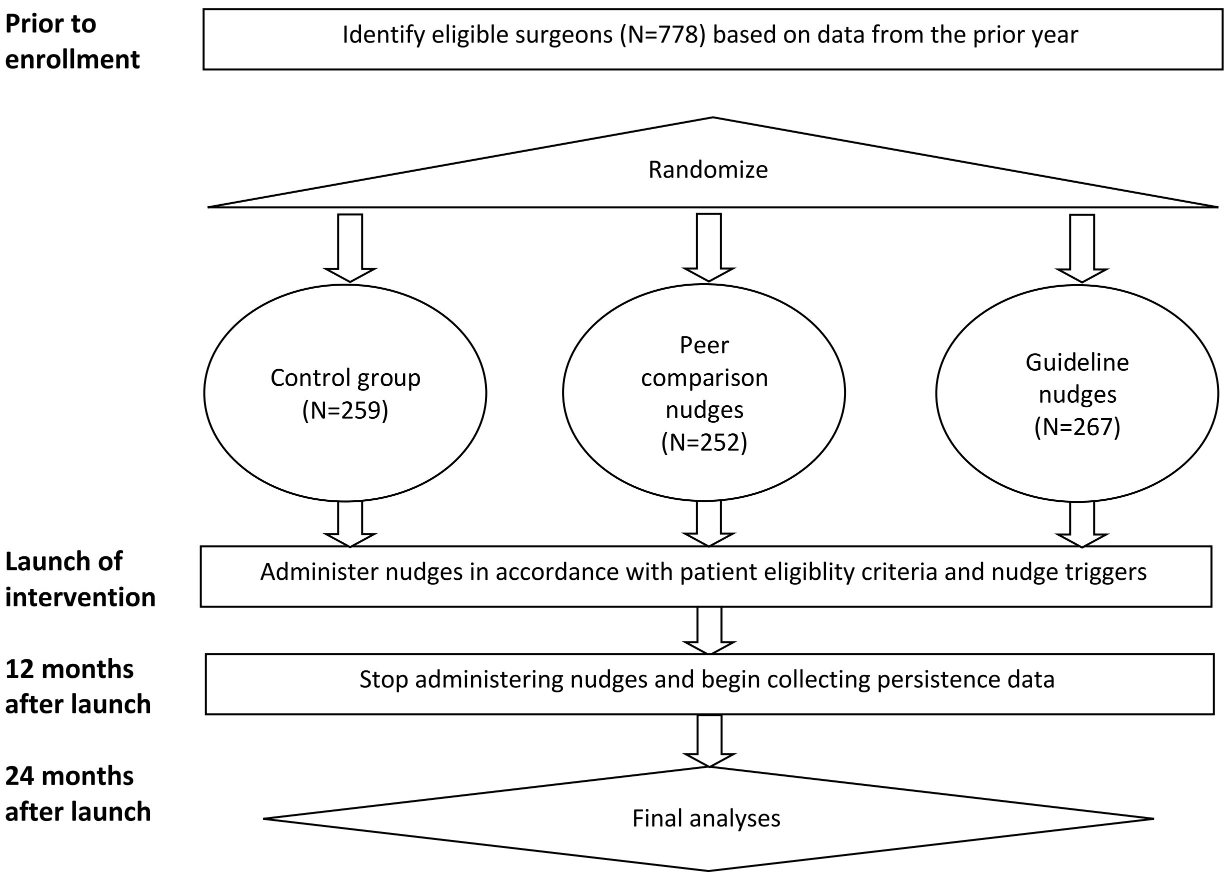

1.3 SCHEDULE OF ACTIVITIES (SOA)

Nudges will be delivered on the third Tuesday of each month for 12 months, October 2021 through September 2022. Data will be collected continuously, with data from the first through last day of the previous calendar month informing whether a nudge is triggered and, if so, the content of the nudge.

After 12 months, the nudges will stop, but data will be collected continuously for another 12 months to assess the persistence of any effects of the nudges.

2 INTRODUCTION

2.1 STUDY RATIONALE

Physician opioid prescribing practices are a major upstream contributor to increases in chronic opioid use, opioid addiction and overdose deaths. Opioid prescribing after surgery is an important intervention target, as opioids are commonly overprescribed after surgery, post-operative opioid prescribing is a risk factor for chronic opioid use, and unused opioids prescribed for patients after surgery are an important reservoir of opioids available for misuse and diversion.

2.2 BACKGROUND

Despite high awareness of the opioid epidemic, clinicians still overprescribe opioids after surgery.[1-7] This post-operative overprescribing puts both patients and communities at risk, increasing the patient's likelihood of developing chronic opioid use[8-14] or opioid-induced ventilatory impairmenINT1 and adding to the reservoir of unused opioids available for misuse and diversion.[11, 15]

The discrepancy between clinicians' awareness of the opioid epidemic and the degree of overprescribing—over half of opioid pills prescribed after surgery go unused[7]—suggests that prescribing practices are not based on purely rational decisions. Indeed, behavioral research has shown that judgment and decision making of both laypeople and experts in a variety of disciplines falls short of rational standards in systematic and predictable ways.[16-19] Even well-informed clinicians make cognitive errors when estimating the benefits and harms of treatment, and these errors are especially likely where there is uncertainty about risks and benefits (as with opioid prescribing decisions for individual patients).[20]

In recent years, behavioral economists and experimental psychologists have successfully leveraged behavioral insights to design "choice architecture" that "nudges" individuals to make better decisions without infringing on their freedom of choice.[21, 22] Such behavioral nudges are promising strategies for changing clinician prescribing behavior because they are often more cost-effective than traditional interventions,[23] can be integrated into existing clinical workflows, and are rapidly scalable once built.

One powerful type of behavioral nudge relies on the strong motivation that most people have to conform with their peers' behavior.[24, 25] Abundant research has found that people (including clinicians) are strongly motivated to adhere to prevailing social norms,[24, 25] and that nudges based on describing social norms can be used to influence prescribing decisions.[26]

Another type of behavioral nudge relies on motivation to follow injunctive norms—to do what is considered the "right thing to do". For example, clinicians may be motivated to follow best practice guidelines published by a well-respected organization. Previous studies suggest that such guidelines are in reality often ignored and thus ineffective in changing behavior,[27, 28] but there is insufficient evidence to determine whether they are more or less effective than nudges that describe peer behavior.

Both of these types of nudges—nudges based on descriptive norms and nudges based on injunctive norms—have been applied to the issue of excessive post-operative opioid prescribing.[29-38] The results have been promising, but because most of these studies have used a pre-post design, it is possible that the observed decreases in prescribing can be explained by a secular trend. Furthermore, all of these studies have bundled and tested different interventions together (e.g., grand rounds presentations or

patient education in addition to nudges), making the effectiveness of the nudges alone unclear. Accordingly, the evidence base for the effectiveness of behavioral nudges in influencing post-operative opioid prescribing is limited.

2.3 RISK/BENEFIT ASSESSMENT

2.3.1 KNOWN POTENTIAL RISKS

We believe that the risk presented by our nudge interventions is negligible, both because the nudges do not prevent the clinicians from using their own clinical judgment and because previous studies have found that reducing the amount of opioids prescribed after surgical operations did not affect patient satisfaction[39, 40], pain scores[39-41], or refill rates[42-44].

2.3.2 KNOWN POTENTIAL BENEFITS

By simultaneously addressing both opioid overprescribing and the science of provider behavior change, we both improve health and advance science. From a public health perspective, overprescribing is the major upstream driver of increases in chronic opioid use and opioid misuse (including the transition to heroin), and studies similar to ours have been successful in curbing this overprescribing.[29-38] From a scientific perspective, examining the comparative effectiveness of ways to operationalize and implement norms, and to examine how this varies across provider types and settings, is at the cutting edge of advances in the science of applied behavioral health economics. Research addressing both issues has the potential for high public health and scientific impact.

2.3.3 ASSESSMENT OF POTENTIAL RISKS AND BENEFITS

We believe that our study's significant potential benefits, especially in the current context of the opioid epidemic, far outweigh the minor and unlikely risks it may present to patients.

3 OBJECTIVES AND ENDPOINTS

OBJECTIVES	ENDPOINTS
Primary	
To test the effectiveness of two behavioral nudges, one based on peer behavior and one based on best practice guidelines, in reducing the share of discharge opioid prescriptions above procedure-specific prescribing guidelines	Share of discharge opioid prescriptions above prescribing guidelines
Secondary	
To test the effectiveness of two behavioral nudges, one based on peer behavior and one based on best practice guidelines, in reducing the morphine milligram equivalent quantities and days' supply prescribed at discharge	Morphine milligram equivalents (MMEs) prescribed at discharge, days' supply of opioids prescribed at discharge
To test the effectiveness of two behavioral nudges, one based on peer behavior and one	Share of discharges where any opioid was prescribed

OBJECTIVES	ENDPOINTS
based on best practice guidelines, in reducing the share of discharges with any opioids prescribed	
To test the long-term effects on patients of two behavioral nudges, one based on peer behavior and one based on best practice guidelines	Share of patients on opioids for greater than 3 months post-discharge
To test the persistence of the effectiveness of two behavioral nudges, one based on peer behavior and one based on best practice guidelines, in reducing the share of discharge opioid prescriptions above procedure-specific prescribing guidelines	Share of discharge opioid prescriptions above prescribing guidelines in the 12 months after the nudges stop being delivered
To test the safety of two behavioral nudges, one based on peer behavior and one based on best practice guidelines	Number of 30-day all-cause emergency department visits, number of 30-day all-cause hospitalizations

4 STUDY DESIGN

4.1 OVERALL DESIGN

We will conduct a three-arm cluster randomized controlled trial of two behavioral nudges compared to usual post-surgical care. One nudge will provide feedback on the surgeon's prescribing behavior relative to institutional prescribing guidelines (an injunctive norm); the other will provide feedback on their prescribing behavior relative to their peers (a descriptive norm). Three surgical specialties (general surgery, orthopedic surgery, and obstetric/gynecological surgery) within 19 hospitals will be randomized such that all surgeons within a given specialty at a given hospital will receive one of three conditions: control, guideline-based nudge, or peer-based nudge.

4.2 SCIENTIFIC RATIONALE FOR STUDY DESIGN

We chose a superiority design in order to make the strongest possible conclusions about whether our nudges are effective and support a policy recommendation as to whether healthcare systems should invest resources in implementing similar nudges.

In our control arm, surgeons will not receive any nudges and will not be informed of the study. By not informing them of the study, we will prevent a Hawthorne effect and obtain an accurate representation of status quo prescribing behavior against which to test the effects of the nudges.

4.3 JUSTIFICATION FOR DOSE

Using prescribing data from recent years at Sutter Health, we simulated how many surgeons would receive nudges and how many nudges they would receive under different study design parameters. From this we determined that delivering the nudges monthly achieved a balance between exposing them to the intervention and burdening them with emails. We chose to deliver the nudges by email because this method presents a low burden to surgeons, has been successful in a similar study conducted at Sutter Health,[45] and would be easily scalable beyond Sutter Health if our nudges prove effective.

4.4 END OF STUDY DEFINITION

The study is considered to be completed once data has been collected for one year after the date on which the final nudges were delivered.

5 STUDY POPULATION

5.1 INCLUSION CRITERIA

Eligible discharge prescriptions meet all of the following criteria:

- the patient is at least 18 years old at the date of surgery
- the patient is discharged to their home
- the surgical procedure has an applicable post-operative opioid prescribing guideline
- the surgical procedure is the only surgical procedure performed during the patient's hospital stay
- the prescription is for an opioid taken orally (tablets, capsules, or liquid solution)

5.2 EXCLUSION CRITERIA

To avoid contamination between the intervention arms, surgeons who operate across multiple surgical specialties (defined as surgeons who performed less than 90% of their total procedures in one specialty between 1 June 2020 and 31 May 2021) will not be eligible.

5.3 LIFESTYLE CONSIDERATIONS

Not applicable.

5.4 SCREEN FAILURES

Not applicable.

5.5 STRATEGIES FOR RECRUITMENT AND RETENTION

We will enroll surgeons by screening all surgeons present in electronic health record data from 1 June 2020 through 31 May 2021, and a waiver of informed consent has been granted by the RAND IRB, so surgeon-level recruitment is unnecessary. Hospitals have been recruited and will be retained through communication with hospital- and system-level administrators.

6 STUDY INTERVENTION

6.1 STUDY INTERVENTION(S) ADMINISTRATION

6.1.1 STUDY INTERVENTION DESCRIPTION

Surgeons randomized to our study intervention will receive one of two types of behavioral nudges delivered as monthly emails. The two nudges will be active for twelve months (October 2021–October 2022).

To ensure that the nudges target only *inappropriate* opioid prescribing, surgeons will receive nudges only when they write opioid prescriptions that exceed post-operative opioid prescribing guidelines developed by multidisciplinary teams at the Mayo Clinic.[32, 46, 47](and personal communication with Professor Elizabeth Habermann, Ph.D., MPH, on opioid prescribing guidelines for caesarean section, March 12, 2021; unreferenced) These guidelines recommend ranges of 5mg oxycodone tablet quantities specific to the procedure performed and are partly based on patient surveys of actual post-operative opioid use.

In both nudge conditions, eligibility for receiving a monthly nudge is contingent upon at least two of the surgeon's patients being discharged with a post-operative opioid prescription exceeding the quantities specified by the Mayo Clinic guidelines. Though it may seem counterintuitive for the descriptive norm nudge to be based implicitly on prescribing guidelines, this choice ensures patient safety and avoids confounding the content of the nudge with the threshold for receiving a nudge.

Intervention arm 1: nudge based on descriptive norms
Surgeons randomized to this condition will receive an email with the following content at the end of each month in which at least two of their patients are discharged with a post-operative opioid prescription that exceeds the prescribing guideline for the procedure performed.

> [Subject line: Your peers vs. your opioid prescribing safety record]
>
> Dear Dr. [Name],
>
> In an effort to reduce opioid use among our surgical patients, Sutter Health is reviewing opioid prescriptions and prescribing patterns for surgeons and will be communicating the findings.
>
> In [month], at least XX of your patients were discharged with opioid prescriptions exceeding the amount prescribed by YY% of your peers for these procedures.
>
> YY% of [specialty] surgeons at Sutter Health prescribe within the ranges below.
>
> We will continue to send you opioid prescribing safety reports.
>
> Sincerely,
> [Signature(s) of chief medical executive, chief of staff, and/or surgical department chair at the surgeon's hospital]
>
> [Table including each procedure type performed by this surgeon in the reference month and the corresponding "Amount prescribed by your peers (5mg oxycodone tablets)", with a footnote stating the conversion factors for hydrocodone and tramadol]

The ranges of 5mg oxycodone tablets displayed in the email will be the same as the ranges stipulated by the prescribing guidelines, but this nudge will not include any language about guidelines.

Intervention arm 2: nudge based on injunctive norms
This condition will be identical to the first condition, except the content of the monthly emails will refer to safety guidelines rather than the surgeon's peers.

[Subject line: Best practice guidelines vs. your opioid prescribing safety record]

Dear Dr. [Name],

In an effort to reduce opioid use among our surgical patients, Sutter Health is reviewing opioid prescriptions and prescribing patterns for surgeons and will be communicating the findings.

In [month], at least XX of your patients were discharged with opioid prescriptions exceeding the amounts recommended by safety guidelines for these procedures.

For patient safety, Sutter Health recommends prescribing within the ranges below for these procedures. Doing so will also meet best practice safety guidelines for post-operative opioid prescribing.

We will continue to send you opioid prescribing safety reports.

Sincerely,
[Signature(s) of chief medical executive, chief of staff, and/or surgical department chair at the surgeon's hospital]

[Table including each procedure type performed by this surgeon in the reference month and the corresponding "Amount recommended by Sutter Health (5mg oxycodone tablets)", with a footnote stating the conversion factors for hydrocodone and tramadol]

Control arm
Surgeons randomized to the control arm will not receive any nudges and will not be informed of the study. By not informing them of the study, we will prevent a Hawthorne effect and obtain an accurate representation of status quo prescribing behavior against which to test the effects of the nudges.

6.1.2 DOSING AND ADMINISTRATION

Nudges will be delivered monthly through the email software MyEmma.

6.2 PREPARATION/HANDLING/STORAGE/ACCOUNTABILITY

6.2.1 ACQUISITION AND ACCOUNTABILITY

Not applicable.

6.2.2 FORMULATION, APPEARANCE, PACKAGING, AND LABELING

Not applicable.

6.2.3 PRODUCT STORAGE AND STABILITY

Not applicable.

6.2.4 PREPARATION

The monthly nudges will be prepared by a project coordinator at Sutter Health using data extracted from Sutter Health's electronic health record by a data analyst at Sutter Health. They will be prepared by

filling in an email template designed and approved by all study investigators. Code used to produce the list of nudge recipients in each month and the information used to fill in the nudge email template (number of discharges with prescriptions above guidelines, procedures for which the receiving surgeon overprescribed, and percent of surgeons in the receiving surgeon's specialty who were compliant with guidelines) has undergone a quality assurance process in which two investigators independently developed code and compared results. The email software MyEmma will be used to prepare and send the nudges.

6.3 MEASURES TO MINIMIZE BIAS: RANDOMIZATION AND BLINDING

The study design has four levels: patients, surgeons, surgical specialties, and hospitals. Randomization will take place at the level of the surgical specialty, using a blocked scheme to ensure that each arm has a balance of large and small hospitals and a sample size of surgeons similar to the other two arms.

All investigators except the Sutter Health data analyst extracting the electronic health record data and the Sutter Health project coordinator preparing the nudges will be blinded to intervention assignment.

6.4 STUDY INTERVENTION COMPLIANCE

A project coordinator at Sutter Health will prepare and send the monthly nudges according to the predetermined schedule (sent on the third Tuesday each month from October 2021 through September 2022). This study team member will report to the site principal investigator, Xiaowei Sherry Yan, and the study principal investigator, Katherine Watkins, after each nudge is sent.

6.5 CONCOMITANT THERAPY

Not applicable.

7 STUDY INTERVENTION DISCONTINUATION AND PARTICIPANT DISCONTINUATION/WITHDRAWAL

7.1 DISCONTINUATION OF STUDY INTERVENTION

The study's independent Data Safety Monitoring Board (DSMB) may make a recommendation to discontinue the study intervention at any time based on an assessment of the benefit/risk ratio of study participation. If the study intervention is discontinued, data will continue to be collected for another 12 months from the date of discontinuation.

7.2 PARTICIPANT DISCONTINUATION/WITHDRAWAL FROM THE STUDY

Participants may discontinue the study intervention at any time by contacting the investigators (an email address will be provided in each nudge) or by clicking the "Unsubscribe" button at the bottom of any nudge email. Their discontinuation and any reasons given will be recorded by the investigators. Data on their prescribing behavior will still be passively collected through the electronic health record until the study's data collection period ends.

7.3 LOST TO FOLLOW-UP

Not applicable.

8 STUDY ASSESSMENTS AND PROCEDURES

8.1 EFFICACY ASSESSMENTS

All efficacy assessments will be conducted using data collected from the Sutter Health electronic health record. Data collected will include:

- Unique study IDs of the patient, hospital location, procedural case record, hospitalization, opioid order, performing provider, discharging provider, and prescribing provider
- Patient sex, racial identity, ethnic identity, and age
- Patient tobacco use and chronic opioid use
- Patient diabetes, substance use disorder, mood disorder, and pain disorder diagnoses
- Patient BMI and ASA physical status rating
- Patient insurance status
- Procedure name, classification (Elective, Emergent, Return to OR, Urgent, Trauma), service line, date, start time, and completion time
- Hospital admission and discharge date and time
- Performing provider title, specialty, gender, medical school graduation year, and board certifications
- Discharge provider title, specialty, gender, medical school graduation year, and board certifications
- Prescribing provider title, specialty, gender, medical school graduation year, and board certifications
- Presence of any opioid prescriptions in the 30 days and 12 months prior to surgery
- Name, quantity, strength, route, date, and time of opioids prescribed in the 24 hours prior to discharge
- Discharge opioid prescription order date, start date, end date, discontinue date, generic name, brand name, strength, quantity, days' supply, class, and refills
- Non-opioid discharge prescription order date, start date, end date, discontinue date, generic name, brand name, strength, and quantity
- Dates and associated diagnoses of emergency department and inpatient visits within 30 days of discharge
- Discharge opioid prescription refills

8.2 SAFETY AND OTHER ASSESSMENTS

All safety assessments will be conducted using data collected from the Sutter Health electronic health record. Data collected will include:

- Dates and associated diagnoses of emergency department visits within 30 days of discharge
- Discharge opioid prescription refills

The study statistician will regularly create three reports for each outcome. Each report will feature a set of ratios indicating the relative frequency of adverse events in a different context. The reports will contain the numerator, denominator, and ratio, each individually reported. Reports will be produced at the end of each month of the trial, including through the 1-year post-intervention period. They will be presented and discussed at half-yearly DSMB meetings.

- Report 1: Adverse events per arm
    a) Numerator: number of adverse events among intervention arm 1 (INT1) patients
    Denominator: total number of INT1 patients meeting the inclusion criteria

b) Numerator: number of adverse events among intervention arm 2 (INT2) patients
Denominator: total number of INT2 patients meeting the inclusion criteria
c) Numerator: number of adverse events among the control patients
Denominator: total number of control patients meeting the inclusion criteria

- Report 2: Adverse events per arm among patients of surgeons who ever received a treatment email
    a) Numerator: number of adverse events among patients whose surgeon ever received an INT1 email
    Denominator: total number of patients whose surgeon ever received an INT1 email
    b) Numerator: number of adverse events among patients whose surgeon ever received an INT2 email
    Denominator: total number of patients whose surgeon ever received an INT2 email

  Notes: Ever received an INT1/INT2 email means that the surgeon is included if they received an INT1/INT2 email in any month of the trial. We will also compare the report 2 ratios to the report 1 control ratio (item c above) for additional context.
- Report 3: Adverse events per arm among patients who were prescribed an opioid at discharge and whose surgeon who ever received a treatment email
    a) Numerator: number of adverse events among patients prescribed opioids at discharge and whose surgeon ever received an INT1 email
    Denominator: total number of patients prescribed opioids at discharge and whose surgeon ever received an INT1 email
    b) Numerator: number of adverse events among patients prescribed opioids at discharge and whose surgeon ever received a nINT2 email
    Denominator: total number of patients prescribed opioids at discharge and whose surgeon ever received an INT2 email
    c) Numerator: number of adverse events among control patients prescribed opioids at discharge
    Denominator: total number of control patients prescribed opioids at discharge

  Notes: Ever received an INT1/INT2 email means that the surgeon is included if they received an INT1/INT2 email in any month of the trial.

8.3 ADVERSE EVENTS AND SERIOUS ADVERSE EVENTS

8.3.1 DEFINITION OF ADVERSE EVENTS (AE)

Adverse event means any untoward medical occurrence associated with the use of an intervention in humans, whether or not considered intervention-related (21 CFR 312.32 (a)).]

8.3.2 DEFINITION OF SERIOUS ADVERSE EVENTS (SAE)

An adverse event (AE) or suspected adverse reaction is considered “serious” if, in the view of either the investigator or sponsor, it results in any of the following outcomes: death, a life-threatening adverse event, or a persistent or significant incapacity or substantial disruption of the ability to conduct normal life functions. Important medical events that may not result in death, be life-threatening, or require hospitalization may be considered serious when, based upon appropriate medical judgment, they may jeopardize the participant and may require medical or surgical intervention to prevent one of the outcomes listed in this definition.

8.3.3 CLASSIFICATION OF AN ADVERSE EVENT

8.3.3.1 SEVERITY OF EVENT

For adverse events (AEs) not included in the protocol defined grading system, the following guidelines will be used to describe severity.

- **Mild** – Events require minimal or no treatment and do not interfere with the participant's daily activities.
- **Moderate** – Events result in a low level of inconvenience or concern with the therapeutic measures. Moderate events may cause some interference with functioning.
- **Severe** – Events interrupt a participant's usual daily activity and may require systemic drug therapy or other treatment. Severe events are usually potentially life-threatening or incapacitating. Of note, the term "severe" does not necessarily equate to "serious".

8.3.3.2 RELATIONSHIP TO STUDY INTERVENTION

All adverse events (AEs) must have their relationship to study intervention assessed by the medical professionals on the study team based on temporal relationship and their clinical judgment. The degree of certainty about causality will be graded using the categories below.

- **Related** – The AE is known to occur with the study intervention, there is a reasonable possibility that the study intervention caused the AE, or there is a temporal relationship between the study intervention and event. Reasonable possibility means that there is evidence to suggest a causal relationship between the study intervention and the AE.
- **Not Related** – There is not a reasonable possibility that the administration of the study intervention caused the event, there is no temporal relationship between the study intervention and event onset, or an alternate etiology has been established.

8.3.3.3 EXPECTEDNESS

The medical professionals on the study team will be responsible for determining whether an adverse event (AE) is expected or unexpected. An AE will be considered unexpected if the nature, severity, or frequency of the event is not consistent with the risk information previously described for the study intervention.

8.3.4 TIME PERIOD AND FREQUENCY FOR EVENT ASSESSMENT AND FOLLOW-UP

The occurrence of an adverse event (AE) or serious adverse event (SAE) may come to the attention of study personnel during the preparation of monthly adverse event reports or half-yearly presentations to the DSMB.

All AEs will be recorded. Information to be collected includes event description, time of onset, clinician's assessment of severity, and relationship to study. All AEs will be followed to adequate resolution.

8.3.5 ADVERSE EVENT REPORTING

All adverse events will be reported to the DSMB at the subsequent DSMB meeting.

8.3.6 SERIOUS ADVERSE EVENT REPORTING

The principal investigator will immediately report to the sponsor and DSMB any serious adverse event, whether or not considered study intervention related, and must include an assessment of whether there is a reasonable possibility that the study intervention caused the event.

8.3.7 REPORTING EVENTS TO PARTICIPANTS
Not applicable.

8.3.8 EVENTS OF SPECIAL INTEREST
Not applicable.

8.3.9 REPORTING OF PREGNANCY
Not applicable.

8.4 UNANTICIPATED PROBLEMS

8.4.1 DEFINITION OF UNANTICIPATED PROBLEMS (UP)
The Office for Human Research Protections (OHRP) considers unanticipated problems involving risks to participants or others to include, in general, any incident, experience, or outcome that meets **all** of the following criteria:

- Unexpected in terms of nature, severity, or frequency given (a) the research procedures that are described in the protocol-related documents, such as the Institutional Review Board (IRB)-approved research protocol and informed consent document; and (b) the characteristics of the participant population being studied;
- Related or possibly related to participation in the research ("possibly related" means there is a reasonable possibility that the incident, experience, or outcome may have been caused by the procedures involved in the research); and
- Suggests that the research places participants or others at a greater risk of harm (including physical, psychological, economic, or social harm) than was previously known or recognized.

8.4.2 UNANTICIPATED PROBLEM REPORTING
The investigators will report unanticipated problems (UPs) to the reviewing Institutional Review Board (IRB). The UP report will include the following information:

- Protocol identifying information: protocol title and number, PI's name, and the IRB project number;
- A detailed description of the event, incident, experience, or outcome;
- An explanation of the basis for determining that the event, incident, experience, or outcome represents an UP;
- A description of any changes to the protocol or other corrective actions that have been taken or are proposed in response to the UP.

To satisfy the requirement for prompt reporting, UPs will be reported using the following timeline:

- UPs that are serious adverse events (SAEs) will be reported to the IRB within <insert timeline in accordance with policy> of the investigator becoming aware of the event.
- Any other UP will be reported to the IRB and to the DCC/study sponsor within <insert timeline in accordance with policy> of the investigator becoming aware of the problem.
- All UPs should be reported to appropriate institutional officials (as required by an institution's written reporting procedures), the supporting agency head (or designee), and the Office for Human Research Protections (OHRP) within <insert timeline in accordance with policy> of the IRB's receipt of the report of the problem from the investigator.]

8.4.3 REPORTING UNANTICIPATED PROBLEMS TO PARTICIPANTS

Not applicable.

9 STATISTICAL CONSIDERATIONS

9.1 STATISTICAL HYPOTHESES

The null hypotheses are that (1) $\beta_1 = 0$, (2) $\beta_2 = 0$, and (3) $\beta_1 = \beta_2$ in the hierarchical linear model

$$Y_{ipsh} = \beta_0 + \beta_1 ARM1_{sh} + \beta_2 ARM2_{sh} + \gamma_{1h} ARM1_{sh} + \gamma_{2h} ARM2_{sh} + \omega_1 X_{ipsh} + \omega_2 Z_{psh} + \omega_3 U_{sh} + \omega_4 W_h + \gamma_h + \eta_{sh} + \varphi_{psh} + \varepsilon_{ipsh}, \quad (1)$$

where i is the patient treated by surgeon p in specialty s at hospital h, $ARM1_{sh}$ and $ARM2_{sh}$ are indicator variables for whether specialty s at hospital h were assigned to intervention arm one or two respectively, X is a set of patient covariates, Z is a set of surgeon covariates, U is a set of specialty covariates, and W is a set of hospital covariates.

The alternative hypotheses are that (1) $\beta_1 \neq 0$, (2) $\beta_2 \neq 0$, and (3) $\beta_1 \neq \beta_2$.

9.2 SAMPLE SIZE DETERMINATION

Statistical power to identify effects of the nudges was examined using recent past data from the participating hospitals. We estimated design parameters required by the PowerUpR package in R software,[48] which provides the capability to estimate statistical power for randomized block clustered designs. Examining medication dose, input parameters for the calculation included unconditional intracluster correlations (ICC) for the hospital (ICC=0.005), service line (ICC=0.039), and provider (ICC=0.337) levels; the number of service line groups (up to three per hospital); the number of providers by service line expected to participate in the study; and number of patients per service line. The ICCs were empirically determined from our preliminary data. We assumed that covariates informative of the dosage would explain between 25 percent and 50 percent of the dosage variation at each of the patient, provider, and service line levels (i.e., R^2 between 0.25 and 0.50). We derived statistical power, assuming one third of the service line groups within hospital will be randomly assigned to each study arm (two treatment and one control). We computed power for pairwise comparison of each of the two nudge arms versus the no nudge arm and adjusted our alpha level to account for multiple comparisons (alpha=0.05/2). We will have 80% power to detect significant differences between the intervention conditions of at least a minimum detectable effect size (MDES) = 0.347 standard deviations (SDs) when R^2=0.25, while R^2=0.5 would yield a MDES of 0.305.

9.3 POPULATIONS FOR ANALYSES

Our main analyses will be conducted on an intention-to-treat dataset including all randomized surgeons. Sensitivity analyses may be conducted on a dataset excluding surgeons who received no nudges.

9.4 STATISTICAL ANALYSES

9.4.1 GENERAL APPROACH

The effects of each nudge intervention relative to the control group and to each other will be tested using a four-level hierarchical model adjusted for multiple hypothesis testing.

9.4.2 ANALYSIS OF THE PRIMARY AND SECONDARY EFFICACY ENDPOINTS

Our primary outcome is the share of discharge prescriptions that were above the guideline for the respective procedure (see above for how guidelines were identified). Prescribing above guidelines is the outcome to which both nudges are linked (even though the descriptive norm nudge does not explicitly refer to guidelines) and thus a key measure of whether clinician behavior responds to the nudges. We define a prescription as being above guidelines if the morphine milligram equivalent (MME) quantity of opioids prescribed is above the ceiling for the procedure-specific guideline (guidelines range from zero to a ceiling). If no opioid is prescribed at discharge, we will code this as within guideline.

We will also analyze the following secondary outcomes to further understand the effects of the intervention.

- MMEs prescribed at discharge
- Days' supply of opioids prescribed at discharge
- Share of discharges where any opioid was prescribed
- Share of patients on opioids for greater than three months post-discharge
- Number of 30-day all-cause emergency department visits
- Number of 30-day all-cause hospitalizations
- Share of discharge opioid prescriptions above prescribing guidelines in the 12 months after the nudges end

Primary analysis

We will analyze outcomes at the level of the discharge using a four-level hierarchical linear model (HLM),[42] thus capturing the clustering inherent in the study design and data generating process. We will analyze outcomes at the patient level, and patients are nested within surgeons, who are nested within specialties, which are nested within hospitals. Both primary and secondary outcomes will follow this modeling structure. To improve the precision of our estimates, we will also include a set of observable patient covariates (X), surgeon covariates (Z), specialty covariates (U), and hospital covariates (W). For patient i, treated by surgeon p, in specialty s, at hospital h, we consider the following HLM formulation for continuous outcomes Y_{ipsh}:

$$\begin{aligned} Y_{ipsh} = \beta_0 + \beta_1 ARM1_{sh} + \beta_2 ARM2_{sh} + \gamma_{1h} ARM1_{sh} + \gamma_{2h} ARM2_{sh} + \\ \omega_1 X_{ipsh} + \omega_2 Z_{psh} + \omega_3 U_{sh} + \omega_4 W_h + \gamma_h + \eta_{sh} + \varphi_{psh} + \varepsilon_{ipsh} \end{aligned} \quad (1)$$

$ARM1_{sh}$ and $ARM2_{sh}$ are indicator variables for whether specialty s, at hospital h were assigned to intervention arm one or two respectively.

The key terms in the equation are $\beta_1$and β_2, the covariate-adjusted treatment effects of arms 1 and 2 relative to the control arm; β_1 answers research question 1 and β_2 answers research question 2. We will use an F-test to compare coefficients β_1 and β_2 to answer research question 3. Thus, the effect of each nudge is estimated relative to receiving no nudges and to the other nudge.

The model allows for the possibility that the treatment effect varies across hospitals, as captured by the random effects $(\gamma_{1h}, \gamma_{2h})$. Unexplained variation in each of the levels is captured by the random effects ε_{ipsh}, φ_{psh}, η_{sh}, and γ_h. We will initially model these six random effects as independent but will also investigate whether including a covariance structure across these components is appropriate. The coefficients ω_1, ω_2, and ω_3, capture the influence of the covariates at the patient, surgeon, and specialty respectively, and covariates will be mean centered as appropriate to aid in model

interpretation. Covariates may include but are not limited to the following: Level 1: patient age, patient sex, patient comorbidities, procedure type, length of operating time; Level 2: surgeon sex, year of surgeon's medical degree; Level 3: total volume of procedures within the specialty: Level 4: number of beds, urbanicity, proportion of patients on Medicaid. Given that the covariates will not change the estimate of the treatment effect (in expectation), only reduce unexplained variance, we will choose a final pool of covariates that we find to be predictive the primary outcome. Model estimates of the treatment effects will adjust standard errors for clustering due to the due to clustered assignment of the interventions.

For binary outcomes, we implement a hierarchical generalized linear model by including a logit link for Equation (1). Note that the Level 1 error term ε_{ipsh} is also eliminated. The concatenated model for all four levels with a binary outcome then reduces to:

$$logit(Y_{ipsh}) = \beta_0 + \beta_1 ARM1_{sh} + \beta_2 ARM2_{sh} + \gamma_{1h} ARM1_{sh} + \gamma_{2h} ARM2_{sh} + \omega_1 X_{ipsh} + \omega_2 Z_{psh} + \omega_3 U_{sh} + \omega_4 W_h + \gamma_h + \eta_{sh} + \varphi_{psh} \quad (2)$$

In the binary outcome version, the parameters $\beta_1$and β_2 again identify the treatment effects of arms 1 and 2 relative to the control arm, with interpretation of these parameters adjusted relative to the link function implemented.

Longitudinal analysis
In addition to assessing the treatment effect averaged over the entire 12-month period, we will also analyze treatment effects by month to assess how the treatment effect evolves over time. For this analysis, we will interact study month indicators with the treatment assignment indicators.

Persistence analysis
We will conduct a secondary analysis to examine whether nudge effects persist once the nudges are discontinued. The data will include the RCT data analyzed in the model above, but also data collected for one year post-intervention (the "persistence period"). The analysis model above will be modified by adding an indicator for the RCT period versus persistence period plus interaction terms for period and each nudge to the model. The statistical significance of these interaction terms will be used to assess whether the treatment effect significantly differs post-RCT.

9.4.3 SAFETY ANALYSES

Safety endpoints include:

- Dates and associated diagnoses of emergency department visits within 30 days of discharge
- Discharge opioid prescription refills

The study statistician will regularly analyze these endpoints as outlined in section 8.2.

9.4.4 BASELINE DESCRIPTIVE STATISTICS

Intervention groups will be compared on the proportion of discharge opioid prescriptions above guidelines, proportion of discharges with any opioid prescription, and average discharge opioid prescription quantity during the baseline period (1 June 2020 through 18 October 2021).

9.4.5 PLANNED INTERIM ANALYSES

Any interim analyses conducted during the intervention period will be solely for the purposes of safety monitoring or planning related studies; the intervention will not be altered unless recommended by the study's Data Safety and Monitoring Board.

9.4.6 SUB-GROUP ANALYSES

We will test for heterogeneity in the treatment effect along several domains. Specifically, we will add terms interacting characteristics of the surgeon with each intervention arm and conduct an F-test of the interaction terms for each nudge.

- Specialty: We will also conduct analyses to test whether the response to each nudge varies by surgeons' specialty.
- Volume of surgeries: We will test for heterogeneity by number of surgeries performed over the 12 month study period. We will only include surgeries for which we have guidelines in this count.
- Baseline opioid prescribing: We will categorize surgeons based on the portion of their surgeries in the 12 months prior to the start of the intervention that were above guidelines. We expect that the intervention will have a larger effect for surgeon with a higher share of prescription above guidelines.

9.4.7 TABULATION OF INDIVIDUAL PARTICIPANT DATA

Not applicable.

9.4.8 EXPLORATORY ANALYSES

None planned.

10 SUPPORTING DOCUMENTATION AND OPERATIONAL CONSIDERATIONS

10.1 REGULATORY, ETHICAL, AND STUDY OVERSIGHT CONSIDERATIONS

10.1.1 INFORMED CONSENT

We requested a waiver of informed consent, which was approved by the RAND IRB. Our rationale was as follows:

- The research involves no more than minimal risk to participants.
- The waiver would not adversely affect the rights and welfare of the participants, as the planned interventions are "nudges" and providers will be free to prescribe what they deem appropriate.
- The research could not practicably be done without a waiver of consent. We expect that allowing providers the opportunity to opt out of this trial would compromise the results of this trial by introducing selection bias.

10.1.2 STUDY DISCONTINUATION AND CLOSURE

This study may be temporarily suspended or prematurely terminated if there is sufficient reasonable cause. If the study is prematurely terminated or suspended, the Principal Investigator (PI) will promptly inform the Institutional Review Board (IRB) and sponsor and will provide the reason(s) for the termination or suspension.
Circumstances that may warrant termination or suspension include, but are not limited to:

- Determination of unexpected, significant, or unacceptable risk to participants

- Demonstration of efficacy that would warrant stopping
- Insufficient compliance to protocol requirements
- Data that are not sufficiently complete and/or evaluable
- Determination that the primary endpoint has been met
- Determination of futility

The study may resume if concerns are addressed satisfactorily, as determined by the DSMB and IRB.

10.1.3 CONFIDENTIALITY AND PRIVACY

Only study investigators will have access to patient and surgeon data, and this access will be governed by a data safeguarding plan approved by the RAND IRB to ensure confidentiality and privacy.

10.1.4 FUTURE USE OF STORED SPECIMENS AND DATA

Fully deidentified datasets will be retained and provided to other investigators upon reasonable request. Limited datasets will be destroyed by the investigators no later than the completion of the project (31 May 2024).

10.1.5 KEY ROLES AND STUDY GOVERNANCE

Principal investigator: Katherine Watkins (kwatkins@rand.org)
Study site principal investigator: Xiaowei Sherry Yan (sherry.yan@sutterhealth.org)

10.1.6 SAFETY OVERSIGHT

Safety oversight will be under the direction of a Data and Safety Monitoring Board (DSMB) composed of individuals with the appropriate expertise, including surgery and pain management. Members of the DSMB will be independent from the study conduct and free of conflict of interest. The DSMB will meet semiannually to assess safety and efficacy data on each arm of the study. The DMSB will operate under the rules of an approved charter that will be written and reviewed at the organizational meeting of the DSMB. At this time, each data element that the DSMB needs to assess will be clearly defined.

DSMB members include the following individuals:

- Ali Arab, PhD
- Stephen Rosenfeld, MD
- Najmeh Parisa Sadoughi, MD
- James Wu, MD

10.1.7 CLINICAL MONITORING

Not applicable.

10.1.8 QUALITY ASSURANCE AND QUALITY CONTROL

Data collected from the Sutter Health electronic health record will be checked for quality. Quality checks will include searching for duplicated prescriptions, prescriptions with missing key information, and prescriptions for quantities likely to be erroneous.

The code used to produce the list of nudge recipients in each month and the information used to fill in the nudge email template (number of discharges with prescriptions above guidelines, procedures for which the receiving surgeon overprescribed, and percent of surgeons in the receiving surgeon’s specialty

who were compliant with guidelines) has undergone a quality assurance process in which two investigators independently developed code and compared results.

10.1.9 DATA HANDLING AND RECORD KEEPING

10.1.9.1 DATA COLLECTION AND MANAGEMENT RESPONSIBILITIES

The following data will be collected by investigators at Sutter Health and the RAND Corporation:

- Interview data. The study team will interview Sutter Health providers about their workflows prior to implementation of the nudges and their experiences of the nudges after implementation. Staff at Sutter Health will facilitate arranging the interviews, so we will not be collecting names and contact information for recruitment.
- Electronic health record (EHR) data. This study will analyze data on clinician prescribing behavior, patient demographic data, and patient clinical data extracted from Sutter Health's EHR. Study team members employed by Sutter Health will extract the data from the EHR, remove all elements of PHI except dates and hospital names, and share this limited dataset within the study team by uploading it to a folder within the project's Teamspace extranet site. Study team members may store this data locally on their password-protected institutionally issued computers for the purpose of data analysis, but will destroy all copies of limited datasets no later than the completion of the project (31 May 2024).
- Pharmacy claims and dispensing data. Study team members at Sutter Health will obtain pharmacy claims and dispensing data via Surescripts, a third-party healthcare information technology organization, so that the study can track changes in opioid dispensing and refills.

The principal investigator, Katherine Watkins, has overall responsibility for the safeguarding of all data, and she will have primary responsibility for familiarizing all the project staff with this data safeguarding plan and procedures. She will follow up with project staff at periodic intervals to ensure that they are in compliance with these data safeguarding procedures.

The study site principal investigator, Xiaowei Sherry Yan, will be responsible for ensuring that the Sutter Health EHR data is collected, stored, and shared in accordance with the procedures outlined above and approved by the RAND IRB.

10.1.9.2 STUDY RECORDS RETENTION

Fully deidentified datasets will be retained indefinitely. Limited datasets will be destroyed by the investigators no later than the completion of the project (31 May 2024).

10.1.10 PROTOCOL DEVIATIONS

A protocol deviation is any noncompliance with the clinical trial protocol or International Conference on Harmonisation Good Clinical Practice (ICH GCP). The noncompliance may be either on the part of the participant, the investigator, or the study site staff. As a result of deviations, corrective actions are to be developed by the site and implemented promptly.

These practices are consistent with ICH GCP:

- 4.5 Compliance with Protocol, sections 4.5.1, 4.5.2, and 4.5.3
- 5.1 Quality Assurance and Quality Control, section 5.1.1
- 5.20 Noncompliance, sections 5.20.1, and 5.20.2.

It is the responsibility of the study site principal investigator, Xiaowei Sherry Yan, to use continuous vigilance to identify and report deviations within 5 working days of identification of the protocol deviation, or within 5 working days of the scheduled protocol-required activity. Protocol deviations must be sent to the reviewing Institutional Review Board (IRB) per their policies. The site investigator is responsible for knowing and adhering to the reviewing IRB requirements.

10.1.11 PUBLICATION AND DATA SHARING POLICY

This study will comply with the NIH Data Sharing Policy and Policy on the Dissemination of NIH-Funded Clinical Trial Information and the Clinical Trials Registration and Results Information Submission rule. As such, this trial has been registered at ClinicalTrials.gov (NCT05070338), and results information from this trial will be submitted to ClinicalTrials.gov. In addition, every attempt will be made to publish results in peer-reviewed journals. Data from this study may be requested from other researchers by contacting the principal investigator, Katherine Watkins (kwatkins@rand.org).

10.1.12 CONFLICT OF INTEREST POLICY

Any actual conflict of interest of persons who have a role in the design, conduct, analysis, publication, or any aspect of this trial will be disclosed and managed. Furthermore, persons who have a perceived conflict of interest will be required to have such conflicts managed in a way that is appropriate to their participation in the design and conduct of this trial.

10.2 ABBREVIATIONS

Abbreviation	Meaning
AE	Adverse Event
CFR	Code of Federal Regulations
DSMB	Data Safety Monitoring Board
EHR	Electronic Health Record
GCP	Good Clinical Practice
HLM	Hierarchical Linear Model
ICC	Intracluster Correlation
ICH	International Conference on Harmonisation
IRB	Institutional Review Board
MME	Morphine Milligram Equivalent
NCT	National Clinical Trial
NIH	National Institutes of Health
OHRP	Office for Human Research Protections
PI	Principal Investigator
RCT	Randomized Controlled Trial
SAE	Serious Adverse Event
SOA	Schedule of Activities
UP	Unanticipated Problem

10.3 PROTOCOL AMENDMENT HISTORY

Version	Date	Description of Change	Brief Rationale
1.0.0	14 January 2019	Original protocol approved by RAND IRB	
1.0.1	19 November 2019	Omitted study advisory panel	Study was funded at 30% less than requested, making the panel infeasible, and the study had sufficient other oversight
1.0.2	13 January 2020	Omitted patient pain survey	NIH project officer suggested this change in response to funding constraints; previous research suggests our intervention is unlikely to substantially increase pain
1.1.0	3 April 2020	Omitted 4 of 7 original surgical specialties, omitted 1 of 4 original arms, modified interventions to include guideline-based nudges, expanded patient population to include opioid-exposed patients and outpatients	Three surgical specialties and one intervention arm were omitted due to funding constraints. Guideline-based nudges were added in response to promising newly developed prescribing guidelines. The patient population was expanded in consideration of sample size and the medical judgment of study investigators with expertise in surgery and anesthesia.
1.1.1	12 October 2021	Updated intervention content and triggers	Intervention content and triggers were refined in light of feedback from Sutter Health hospital administrators and behavioral scientists on the study team

11 REFERENCES

1. Dixit AA, Chen CL, Inglis-Arkell C, Manuel SP. Assessment of Unused Opioids Following Ambulatory Surgery. *American Surgeon*. Jun 2020;86(6):652-658. doi:10.1177/0003134820923309

2. Egan KG, De Souza M, Muenks E, Nazir N, Korentager R. Predictors of Opioid Consumption in Immediate, Implant-Based Breast Reconstruction. *Plast Reconstr Surg*. Oct 2020;146(4):734-741. doi:10.1097/PRS.0000000000007150

3. Gunasingha R, Niloy IL, Wetstein BB, Learn PA, Turza LC. Keeping tabs: Reducing postoperative opioid prescriptions for patients after breast surgical procedures. *Surgery*. Jun 2021;169(6):1316-1322. doi:10.1016/j.surg.2020.11.045

4. Kamdar PM, Mandava NK, Narula A, et al. Opioid Use After Knee Arthroscopy. *Arthrosc Sports Med Rehabil*. Apr 2020;2(2):e77-e81. doi:10.1016/j.asmr.2019.11.003

5. Kunkel ST, Sabatino MJ, Pierce DA, Fillingham YA, Jevsevar DS, Moschetti WE. What Happens to Unused Opioids After Total Joint Arthroplasty? An Evaluation of Unused Postoperative Opioid Disposal Practices. *J Arthroplasty*. Apr 2020;35(4):966-970. doi:10.1016/j.arth.2019.11.013

6. Runner RP, Luu AN, Thielen ZP, et al. Opioid Use After Discharge Following Primary Unilateral Total Hip Arthroplasty: How Much Are We Overprescribing? *J Arthroplasty*. Jun 2020;35(6S):S226-S230. doi:10.1016/j.arth.2020.01.076

7. Schirle L, Stone AL, Morris MC, et al. Leftover opioids following adult surgical procedures: a systematic review and meta-analysis. *Syst Rev*. Jun 11 2020;9(1):139. doi:10.1186/s13643-020-01393-8

8. Alam A, Gomes T, Zheng H, Mamdani MM, Juurlink DN, Bell CM. Long-term analgesic use after low-risk surgery: A retrospective cohort study. *Arch Intern Med*. 2012;172(5):425-430.

9. Brummett CM, Waljee JF, Goesling J, et al. New persistent opioid use after minor and major surgical procedures in US adults. *JAMA Surg*. 2017:e170504. doi:10.1001/jamasurg.2017.0504

10. Clarke H, Soneji N, Ko DT, Yun L, Wijeysundera DN. Rates and risk factors for prolonged opioid use after major surgery: Population based cohort study. *BMJ*. 2014;348:g1251.

11. Levy N, Quinlan J, El-Boghdadly K, et al. An international multidisciplinary consensus statement on the prevention of opioid-related harm in adult surgical patients. *Anaesthesia*. Apr 2021;76(4):520-536. doi:10.1111/anae.15262

12. Raebel MA, Newcomer SR, Reifler LM, et al. Chronic use of opioid medications before and after bariatric surgery. *JAMA*. 2013;310(13):1369-1376.

13. Sun EC, Darnall BD, Baker LC, Mackey S. Incidence of and risk factors for chronic opioid use among opioid-naive patients in the postoperative period. *JAMA Intern Med*. 2016;176(9):1286-1293. doi:10.1001/jamainternmed.2016.3298

14. Waljee JF, Li L, Brummett CM, Englesbe MJ. Iatrogenic opioid dependence in the United States: Are surgeons the gatekeepers? *Annals of Surgery*. 2017;265(4):728-730.

15. Bicket MC, Long JJ, Pronovost PJ, Alexander GC, Wu CL. Prescription Opioid Analgesics Commonly Unused After Surgery: A Systematic Review. *JAMA Surg*. Nov 1 2017;152(11):1066-1071. doi:10.1001/jamasurg.2017.0831

16. Gilovich T, Griffin D, Kahneman D. *Heuristics and Biases: The Psychology of Intuitive Judgment*. Cambridge University Press; 2002.

17. Kahneman D, Slovic P, Tversky A. *Judgment under Uncertainty: Heuristics and Biases*. Cambridge University Press; 1982.

18. Kahneman D, Tversky A. *Choices, Values, and Frames*. Cambridge University Press; 2000.

19. Keren G, Wu G. *The Wiley Blackwell Handbook of Judgment and Decision Making*. John Wiley & Sons; 2016.

20. Hoffmann TC, Del Mar C. Clinicians' expectations of the benefits and harms of treatments, screening, and tests: A systematic review. *JAMA Intern Med*. 2017;177(3):407-419.

21. Johnson EJ, Shu SB, Dellaert BG, et al. Beyond nudges: Tools of a choice architecture. *Mark Lett*. 2012;23(2):487-504.

22. Thaler RH, Sunstein CR. *Nudge: Improving Decisions about Health, Wealth, and Happiness*. Penguin Group; 2008.

23. Benartzi S, Beshears J, Milkman KL, et al. Should governments invest more in nudging? *Psychol Sci*. 2017;28(8):1041-1055.

24. Cialdini RB, Goldstein NJ. Social influence: Compliance and conformity. *Annu Rev Psychol*. 2004;55:591-621. doi:10.1146/annurev.psych.55.090902.142015

25. Rogers T, Goldstein NJ, Fox CR. Social Mobilization. *Annu Rev Psychol*. Jan 4 2018;69:357-381. doi:10.1146/annurev-psych-122414-033718

26. Meeker D, Linder JA, Fox CR, et al. Effect of behavioral interventions on inappropriate antibiotic prescribing among primary care practices: A randomized clinical trial. *JAMA*. 2016;315(6):562-570.

27. Alpert JS. Why are we ignoring guideline recommendations? *Am J Med*. Feb 2010;123(2):97-8. doi:10.1016/j.amjmed.2009.10.003

28. Cabana MD, Rand CS, Powe NR, et al. Why don't physicians follow clinical practice guidelines? A framework for improvement. *JAMA*. Oct 20 1999;282(15):1458-65. doi:10.1001/jama.282.15.1458

29. Bongiovanni T, Hansen K, Lancaster E, O'Sullivan P, Hirose K, Wick E. Adopting best practices in post-operative analgesia prescribing in a safety-net hospital: Residents as a conduit to change. *Am J Surg*. Feb 2020;219(2):299-303. doi:10.1016/j.amjsurg.2019.12.023

30. Choo KJ, Grace TR, Khanna K, Barry J, Hansen EN. A Goal-directed Quality Improvement Initiative to Reduce Opioid Prescriptions After Orthopaedic Procedures. *J Am Acad Orthop Surg Glob Res Rev*. 2019;3(9):e109. doi:10.5435/JAAOSGlobal-D-19-00109

31. Fearon NJ, Benfante N, Assel M, et al. Standardizing Opioid Prescriptions to Patients After Ambulatory Oncologic Surgery Reduces Overprescription. *Jt Comm J Qual Patient Saf*. Jul 2020;46(7):410-416. doi:10.1016/j.jcjq.2020.04.004

32. Glaser GE, Kalogera E, Kumar A, et al. Outcomes and Patient Perspectives Following Implementation of Tiered Opioid Prescription Guidelines in Gynecologic Surgery. *Gynecologic Oncology*. May 2020 2020;157(2):476-481. doi:10.1016/j.ygyno.2020.02.025

33. Jacobs BL, Rogers D, Yabes JG, et al. Large reduction in opioid prescribing by a multipronged behavioral intervention after major urologic surgery. *Cancer*. Jan 15 2021;127(2):257-265. doi:10.1002/cncr.33200

34. Kaafarani H, Eid A, Antonelli D, et al. Description and Impact of a Comprehensive Multispecialty Multidisciplinary Intervention to Decrease Opioid Prescribing in Surgery. *Annals of Surgery*. 2019 Sep 2019;270(3)doi:10.1097/SLA.0000000000003462

35. Smalley CM, Willner MA, Muir MR, et al. Electronic medical record-based interventions to encourage opioid prescribing best practices in the emergency department. *American Journal of Emergency Medicine*. Aug 2020;38(8):1647-1651. doi:10.1016/j.ajem.2019.158500

36. Stulberg JJ, Schafer WLA, Shallcross ML, et al. Evaluating the implementation and effectiveness of a multi-component intervention to reduce post-surgical opioid prescribing: study protocol of a mixed-methods design. *BMJ Open*. Jun 3 2019;9(6):e030404. doi:10.1136/bmjopen-2019-030404

37. Zhang DDQ, Sussman J, Dossa F, et al. A Systematic Review of Behavioral Interventions to Decrease Opioid Prescribing After Surgery. *Annals of Surgery*. Feb 2020;271(2):266-278. doi:10.1097/SLA.0000000000003483

38. Ziegelmann M, Joseph J, Glasgow A, et al. Comparison of prescribing patterns before and after implementation of evidence-based opioid prescribing guidelines for the postoperative urologic surgery patient. *American Journal of Surgery*. Aug 2020;220(2):499-504. doi:10.1016/j.amjsurg.2019.11.037

39. Bateman BT, Cole NM, Maeda A, et al. Patterns of Opioid Prescription and Use After Cesarean Delivery. *Obstet Gynecol*. Jul 2017;130(1):29-35. doi:10.1097/AOG.0000000000002093

40. Vu JV, Howard RA, Gunaseelan V, Brummett CM, Waljee JF, Englesbe MJ. Statewide Implementation of Postoperative Opioid Prescribing Guidelines. *New England Journal of Medicine*. Aug 15 2019;381(7):680-682. doi:10.1056/NEJMc1905045

41. Lee JS, Hu HM, Brummett CM, et al. Postoperative Opioid Prescribing and the Pain Scores on Hospital Consumer Assessment of Healthcare Providers and Systems Survey. *JAMA*. May 16 2017;317(19):2013-2015. doi:10.1001/jama.2017.2827

42. Hill MV, Stucke RS, McMahon ML, Beeman JL, Barth RJ, Jr. An Educational Intervention Decreases Opioid Prescribing After General Surgical Operations. *Annals of Surgery*. Mar 2018;267(3):468-472. doi:10.1097/SLA.0000000000002198

43. Lee JS, Howard RA, Klueh MP, et al. The Impact of Education and Prescribing Guidelines on Opioid Prescribing for Breast and Melanoma Procedures. *Ann Surg Oncol*. Jan 2019;26(1):17-24. doi:10.1245/s10434-018-6772-3

44. Sekhri S, Arora NS, Cottrell H, et al. Probability of Opioid Prescription Refilling After Surgery: Does Initial Prescription Dose Matter? *Annals of Surgery*. Aug 2018;268(2):271-276. doi:10.1097/SLA.0000000000002308

45. Navathe A, Liao J, Delgado K, et al. Effect of Peer Comparison Feedback, Individual Audit Feedback or Both to Clinicians on Opioid Prescribing in Acute Care Settings: A Cluster Randomized Clinical Trial. *Health Services Research*. 2021;56(S2):55-56. doi:10.1111/1475-6773.13775

46. Thiels CA, Ubl DS, Yost KJ, et al. Results of a Prospective, Multicenter Initiative Aimed at Developing Opioid-prescribing Guidelines After Surgery. *Annals of Surgery*. 2018 Sep 2018;268(3):457-468. doi:10.1097/SLA.0000000000002919

47. Wyles CC, Hevesi M, Ubl DS, et al. Implementation of Procedure-Specific Opioid Guidelines: A Readily Employable Strategy to Improve Consistency and Decrease Excessive Prescribing Following Orthopaedic Surgery. *JB JS Open Access*. Jan-Mar 2020;5(1):e0050. doi:10.2106/JBJS.OA.19.00050

48. *PowerUpR: Power analysis tools for multilevel randomized experiments*. Version 0.1.3. 2017.
