# Supplementary material for: Peer Comparison or Guideline-Based Feedback and Postsurgery Opioid Prescriptions: A Randomized Clinical Trial
Source: JAMA Health Forum. 2024 Mar 15;5(3):e240077. doi: 10.1001/jamahealthforum.2024.0077 (PMC10943416; doi:10.1001/jamahealthforum.2024.0077)
Supplement: Supplement 2. — eAppendix eTable 1. Prescribing guidelines for procedures included in the present study eTable 2. Intervention impacts over time (full output from Figure 2B in main text) eTable 3. Intervention effects with full set of covariates included eTable 4. Subgroup analyses of intervention impacts on guideline-discordant prescribing eTable 5. Tests for differences in intervention impacts by subgroup eTable 6. Effect of social norms-based nudges on primary and secondary outcomes (discharge level) eTable 7. Intervention effects on patient outcomes eTable 8. Sensitivity analyses using surgeons who were more exposed to the intervention eFigure 1. Example of Peer Comparison Intervention Email eFigure 2. Example of Guidelines Intervention Email eFigure 3. Effect of social norms-based nudges on each quantile of MMEs prescribed at discharge eFigure 4. Exposure to interventions [file jamahealthforum-e240077-s002.pdf]

Supplemental Online Content

Wagner Z, Kirkegaard A, Mariano LT, et al. Peer comparison or guideline-based feedback vs no feedback on postsurgery opioid prescriptions: a randomized clinical trial. *JAMA Health Forum*. 2024;5(3):e240077. doi:10.1001/jamahealthforum.2024.0077

Additional Details on Statistical Analysis

Prespecified Analyses

Primary analysis: Hierarchical linear model

Intervention effects over time

Subgroup analysis

Exploratory Analyses

Discharge level model

Quantile regression

Power Calculations (from study protocol)

eTable 1. Prescribing guidelines for procedures included in the present study

eTable 2. Intervention impacts over time (full output from Figure 2B in main text)

eTable 3. Intervention effects with full set of covariates included

eTable 4. Subgroup analyses of intervention impacts on guideline-discordant prescribing

eTable 5. Tests for differences in intervention impacts by subgroup

eTable 6. Effect of social norms-based nudges on primary and secondary outcomes (discharge level)

eTable 7. Intervention effects on patient outcomes

eTable 8. Sensitivity analyses using surgeons who were more exposed to the intervention

eFigure 1. Example of Peer Comparison Intervention Email

eFigure 2. Example of Guidelines Intervention Email

eFigure 3. Effect of social norms-based nudges on each quantile of MMEs prescribed at discharge

eFigure 4. Exposure to interventions

© 2024 Wagner Z, et al. *JAMA Health Forum.*2024. All rights reserved.

This supplemental material has been provided by the authors to give readers additional information about their work.

© 2024 Wagner Z, et al. *JAMA Health Forum.*2024. All rights reserved.

Additional Details on Statistical Analysis

Prespecified Analyses

Primary analysis: Hierarchical linear model

We modeled intervention effects at the level of the surgical specialty using a hierarchical linear model (HLM).[1] The HLM captures the clustering inherent in the study design and data generating process. Outcomes are at the patient discharge level, and discharged patients are nested within surgeons, who are nested within specialty, which are nested within hospitals. Treatment was assigned at the level of the specialty within hospital. For our primary outcome, prescribing above guidelines, which is binary, our model takes the following form for patient i, treated by surgeon provider p, in specialty s, at hospital h:

$$logit(Y_{ipsh}) = \beta_0 + \beta_1 Peer_{sh} + \beta_2 Guidelines_{sh} + \omega Z_{psh} + \eta_{sh} + \varphi_{psh}$$

$Peer_{sh}$ and $Guidelines_{sh}$ are indicator variables for whether specialty s, at hospital h were assigned to the peer comparison or guidelines arm. These terms enter the model at the level of the specialty within a hospital. The key terms in the equation are $\beta_1$and β_2, the covariate-adjusted treatment effects of the peer comparison and guidelines arms relative to the control arm. Unexplained variation within providers and within specialty are captured by the random effects, φ_{psh}, and η_{sh}. Note that a random effect for the hospital could also be included in the model. For this dataset, we find the small number of specialties per hospital to be insufficient to support inclusion of such an effect. The coefficient, ω captures the influence of the covariates at the surgeon level Z_{psh}, for which we include the baseline measure of the outcome (calculated at the surgeon level using data from the 24 months prior to the intervention). Additional covariates, including those from the patient, specialty, and hospital levels could also be included. The baseline measure of the outcome proved to be highly correlated with the outcome, and additional variables yield little predictive value after inclusion of the baseline measure. Thus, our adjusted models feature the parsimonious choice of just including the baseline outcome measure. We investigated the treatment effects on the secondary outcome of whether any opioids were prescribed using this same modeling structure.

We convert all estimated of intervention effects to the scale of the outcome variable for ease of interpretability and report the absolute percentage points difference in Table 2. The conversion is created using the method of recycled prediction, which is also referred to as predictive margins, using R's *margins* package.[2] Standard errors for the predicted margins were estimated using the delta method.

The secondary outcome of MMEs prescribed at post-surgery discharge follow a similar framework to that presented above, but for a continuous outcome:

$$Y_{ipsh} = \beta_0 + \beta_1 Peer_{sh} + \beta_2 Guidelines_{sh} + \omega Z_{psh} + \eta_{sh} + \varphi_{psh} + \varepsilon_{ipsh}$$

Note that in this version of the model an additional variance component at the patient level in included. The estimated treatment effects $\beta_1$and β_2 are fit on the scale of the outcome.

© 2024 Wagner Z, et al. *JAMA Health Forum.*2024. All rights reserved.

Intervention effects over time
To estimate intervention effects in each of the 12 study months, we ran separate HLMs, identical to the form described above, for each of the 12 study months (12 separate models) and plotted coefficients and 95% confidence intervals in Figure 2b of the main paper and presented results in Table S2.

Subgroup analysis
We estimated separate HLMs for pre-specified subgroups of surgeons based on three surgeon characteristics:

1. *Surgical procedure volume*: We aggregated data to the surgeon level and calculated the number of discharged patients in our data during the intervention for each surgeon. We created 4 categories of surgeons based on the quartiles: 1-13, 14-34, 35-84, >84 discharges.
2. *Baseline guideline-discordant prescribing:* We aggregated data to the surgeon level and calculated the share of discharged patients with an opioid prescription above guidelines in the 24 months prior to intervention. We created 4 categories of surgeons based on the quartiles: 0-8.7%, 8.8-35.7%, 35.8-58.5%, and >58.5% of prescriptions above guidelines.
3. *Surgical specialty:* We used the electronic health records to identify each surgeon's specialty.

We tested for differences in the intervention impact between subgroups scaling the difference between individual subgroup estimates by their pooled standard error. For procedure volume and baseline guideline-discordant prescribing, we combined the top two and bottom two categories of surgeons (1st and 2nd quartile and 3rd and 4th quartile) creating two categories representing above or below the median. This is because we had limited statistical power to test for differences between subgroup when we included four categories.

Exploratory Analyses

Discharge level model
Our primary analytical approach, the HLM, implicitly down-weights observations from surgeons who had higher variance in prescribing. In our data, this tended to be surgeons that performed more discharges.[3] This could result in underestimation of the public health benefits of the intervention because 1) surgeons with more discharges were more impacted by the intervention (see Figure 3 of main text and Table S2), likely because they had more opportunities to receive an email and had more guideline-discordant prescriptions prior to the intervention, and 2) these surgeons affected a larger share of the patient population by definition (i.e. they had more discharges). To estimate average intervention impacts at the discharge-level, we used ordinary least squares (OLS) models controlling for baseline levels of the outcome (mean of the outcome at the surgeon level in the 24 months prior to the intervention). These models weight each discharge equally. We account for the clustered designed in these models by clustering standard

© 2024 Wagner Z, et al. *JAMA Health Forum.*2024. All rights reserved.

errors by specialty within hospital, which is the level at which the intervention was randomly assigned.[4] The model takes the following form:

$$y_{isc} = \beta_0 + \beta_1 Peer_c + \beta_2 Guidelines_c + bl\_y_s + \epsilon_{isc}$$

Where y_{isc} is the outcome for discharged patient i with operating surgeon s working in cluster c (the surgeon's specialty-hospital combination). The $Peer$ and $Guidlines$ terms are indicators for the peer comparison intervention and the guidelines intervention, respectively, and vary only by cluster c. The bl_y term is the mean of the outcome in the 24 months prior to the intervention calculated at the surgeon level. The β_1 and β_2 coefficients are the average intervention impacts per discharge and are what is reported in Table S5.

Quantile regression
We used Stata's -qreg2- command to estimate quantile regressions presented in Figure S4. These models controlled for baseline guideline-discordant prescribing in the same way as in the other models and clustered standard errors by specialty within hospital. We ran separated quantile regressions for each quantile assessed.

Power Calculations (from study protocol)

Statistical power to identify effects of the nudges was examined using recent past data from the participating hospitals. We estimated design parameters required by the PowerUpR package in R software, which provides the capability to estimate statistical power for randomized block clustered designs. Examining medication dose, input parameters for the calculation included unconditional intracluster correlations (ICC) for the hospital (ICC=0.005), service line (ICC=0.039) and provider (ICC=0.337) levels; the number of service line groups (up to three per hospital); the number of providers by service line expected to participate in the study and number of patients per service line. The ICCs were empirically determined from our preliminary data. We assumed that covariates informative of the dosage would explain between 25% and 50% of the dosage variation at each of the patient, provider and service line levels (ie, R^2 between 0.25 and 0.50). We derived statistical power, assuming one-third of the service line groups within hospital will be randomly assigned to each study arm (two treatment and one control). We computed power for pairwise comparison of each of the two nudge arms versus the no nudge arm and adjusted our alpha level to account for multiple comparisons (alpha=0.05/2). We will have 80% power to detect significant differences between the intervention conditions of at least a minimum detectable effect size (MDES)=0.347 SDs when R^2=0.25, while R^2=0.5 would yield an MDES of 0.305. With R^2=0.25, detectible differences in percentage prescribed above guidelines range from 12.0 to 13.8 percentage points for baseline rates of 25% and 50% above guidelines respectively. With R^2=0.5, detectible differences in percentage prescribed above guidelines decrease, ranging from 10.5 to 12.3 percentage points for the same baseline rates.

© 2024 Wagner Z, et al. *JAMA Health Forum.*2024. All rights reserved.

Supplementary Tables

eTable 1. Prescribing guidelines for procedures included in the present study

Procedure	Recommended quantity of 5mg oxycodone tabs
ACL reconstruction	0–25
Acute fracture management	0–15
Ankle arthrodesis	0–40
Ankle fracture ORIF	0–40
Bronchoscopy or upper endoscopy	0
C-section	0–10
Carotid endarterectomy	0
Carpal tunnel release	0–15
Colon or small bowel surgery	0–15
Distal radial ORIF	0–40
Endoscopy	0
Femoral-neck fracture ORIF	0–25
Gynecologic laparotomy (hysterectomy, omentectomy, lymphadenectomy, etc.)	0–5
Hysteroscopy	0
Knee arthroscopy	0–25
Lumbar laminectomy or laminotomy with arthrodesis	0–50
Lumbar laminectomy or laminotomy without arthrodesis	0–40
Major spine surgery	0–50
Mastectomy with subcutaneous reconstruction	0–15
Mastectomy with submuscular reconstruction	0–30
Minimally invasive gynecologic surgery (laparoscopic or robotic)	0–5
Minor spine surgery	0–40
MIS abdominal solid organ resection	0–15

© 2024 Wagner Z, et al. *JAMA Health Forum.*2024. All rights reserved.

MIS bariatric, benign foregut, or adrenal surgery	0–8
MIS cholecystectomy or appendectomy	0–8
MIS inguinal hernia repair	0–8
MTP arthrodesis	0–25
Muscle biopsy or excisional biopsy	0
Open inguinal hernia repair	0–8
Open major abdominal resection	0–30
Percutaneous endovascular or vascular access procedure	0
Shoulder arthroscopy	0–40
Simple mastectomy	0–10
Thoracotomy (pulmonary, pleural, or chest wall)	0–50
Thumb basal joint reconstruction	0–25
Thyroid/parathyroid surgery, mediastinoscopy, or POEM	0–5
Total hip arthroplasty	0–50
Total knee arthroplasty	0–50
Total shoulder arthroplasty	0–50
VATS procedure (pulmonary or mediastinal)	0–20
Wide local excision or lumpectomy	0–5

Source: Mayo Clinic. Quantities are expressed in terms of the total quantity recommended for the postoperative period rather than a duration-specific measure such as days' supply.

© 2024 Wagner Z, et al. *JAMA Health Forum.*2024. All rights reserved.

eTable 2. Intervention impacts over time (full output from Figure 2B in main text)

	Average of Outcome in Each Arm			Effect of Peer Comparison Intervention		Effect of Guidelines Intervention	
	Control	Peer Comparison	Guidelines	Adjusted (95% CI)	p-value	Adjusted (95% CI)	p-value
Month 1	36.3	32.7	27.3	-4.2<br>(-10.0, 1.7)	0.165	-6.4<br>(-12.0, -0.8)	0.050
Month 2	33.3	32.0	22.1	-3.6<br>(-8.7, 1.4)	0.155	-10.1<br>(-14.7, -5.5)	0.000
Month 3	37.7	34.0	27.5	-3.8<br>(-10.2, 2.6)	0.245	-8.3<br>(-14.4, -2.2)	0.016
Month 4	34.8	29.0	28.0	-4.5<br>(-11.3, 2.2)	0.378	-2.6<br>(-9.5, 4.2)	0.455
Month 5	37.3	25.9	25.4	-10.7<br>(-15.9, -5.5)	0.000	-8.3<br>(-13.7, -3.0)	0.002
Month 6	36.9	25.8	27.0	-10.5<br>(-16.4, -4.7)	0.001	-7.8<br>(-13.5, -2.1)	0.007
Month 7	38.1	22.9	27.0	-11.9<br>(-17.9, -5.9)	0.000	-8.1<br>(-14.4, -1.9)	0.010
Month 8	36.6	27.3	22.1	-9.1<br>(-16.2, -2.0)	0.012	-11.9<br>(-18.7, -5.0)	0.001
Month 9	39.8	27.4	26.9	-12.6<br>(-18.5, -6.6)	0.000	-12.0<br>(-18.1, -5.9)	0.000
Month 10	38.9	27.7	24.6	-9.9<br>(-16.2, -3.6)	0.004	-9.1<br>(-16.2, -2.0)	0.012
Month 11	37.9	24.2	24.3	-9.4<br>(-16.6, -2.2)	0.010	-9.3<br>(-16.5, -2.2)	0.010
Month 12	35.2	21.2	22.9	-13.2<br>(-18.6, -7.7)	0.000	-10.4<br>(-15.7, -5.0)	0.000

Adjusted models include random effects for surgeon and specialty within hospital and control for baseline level of the outcome at the surgeon level. P-values are adjusted for multiple testing using a false discovery rate of 0.05.

© 2024 Wagner Z, et al. *JAMA Health Forum.*2024. All rights reserved.

eTable 3. Intervention effects with full set of covariates included

	Effect of Peer Comparison Intervention		Effect of Guidelines Intervention	
Outcome	Adjusted (95% CI)	p-value	Adjusted (95% CI)	p-value
Above guidelines	-6.4 (-9.3, -3.4)	< 0.001	-4.3 (-7.1, -1.4)	0.003
MMEs	-6.3 (-19.7, 7.0)	0.671	-2.5 (-15.6, 10.5)	0.691
Any opioid	0.2 (-4.2, 4.5)	0.930	3.0 (-1.2, 7.2)	0.312

Notes: Estimates are from hierarchical models that include random effects for surgeon and specialty within hospital and control for baseline level of the outcome; provider location; procedure guideline category; service line; operating room case classification; quarter of the year; provider's gender; and the patient's age, ASA score; length of hospital stay; gender, race/ethnicity, Medicaid status, diabetic status, pain status, chronic opioid use, and previous opioid use in the past day, month, and year, . P-values are adjusted for multiple testing using a false discovery rate of 0.05.

© 2024 Wagner Z, et al. *JAMA Health Forum.*2024. All rights reserved.

eTable 4. Subgroup analyses of intervention impacts on guideline-discordant prescribing

	Average of Outcome in Each Arm			Effect of Peer Comparison Intervention		Effect of Guidelines Intervention	
	Control	Peer Comparison	Guidelines	Adjusted (95% CI)	p-value	Adjusted (95% CI)	p-value
Procedure Volume							
1-13 procedures	28.7	14.8	23.7	-1.4	0.721	-3.6	0.697
				(-9.3, 6.4)		(-11.1, 3.9)	
14-34 procedures	31.9	27.8	33.0	-8.6	0.003	-14.0	0.000
				(-14.3, -2.9)		(-19.5, -8.5)	
35-84 procedures	41.0	31.1	24.6	-9.0	0.156	-3.1	0.571
				(-19.1, 1.0)		(-14.0, 7.8)	
>84 procedures	36.8	26.2	24.8	-10.5	0.000	-7.7	0.005
				(-16.0, -4.9)		(-13.1, -2.4)	
Baseline Share Above Guidelines							
0-8.7%	5.0	4.1	3.3	-1.6	0.420	-1.5	0.420
				(-5.0, 1.8)		(-5.0, 2.1)	
8.8-35.7%	17.8	14.1	16.3	-2.1	0.634	-1.0	0.634
				(-6.8, 2.7)		(-5.2, 3.2)	
35.8-58.7%	46.5	33.6	30.9	-10.4	0.003	-13.1	0.000
				(-17.2, -3.6)		(-20.0, -6.2)	
>58.7%	67.6	48.7	48.2	-13.2	0.032	-12.1	0.032
				(-24.5, -1.9)		(-23.3, -1.0)	
**Surgical Specialty**							
General	37.3	33.7	28.5	-4.8	0.159	-3.2	0.240
				(-10.1, 0.6)		(-8.5, 2.1)	
Obstetrics and gynecology	43.6	31.5	28.1	-5.6	0.206	-9.7	0.044
				(-14.3, 3.1)		(-18.0, -1.4)	
Orthopedics	16.4	6.0	19.1	-5.2	0.147	-1.3	0.625
				(-10.8, 0.5)		(-6.7, 4.0)	

Adjusted models include random effects for surgeon and specialty within hospital and control for baseline level of the outcome at the surgeon level. Adjusted coefficients and confidents intervals are plotted in Figure 3 of the main paper. P-values are adjusted for multiple testing using a false discovery rate of 0.05.

© 2024 Wagner Z, et al. *JAMA Health Forum.*2024. All rights reserved.

eTable 5. Tests for differences in intervention impacts by subgroup

	Effect of Peer Comparison Intervention		Effect of Guidelines Intervention	
	Adjusted (95% CI)	p-value	Adjusted (95% CI)	p-value
Procedure Volume				
Below median (1-34 procedures)	-3.4	0.728	-1.0	0.796
	(-10.7, 3.9)		(-8.3, 6.4)	
Above median (>35 procedures)	-8.8	<0.001	-9.5	<0.001
	(-13.0, -4.6)		(-13.8, -5.3)	
Difference in effect	-5.4	0.207	-8.6	0.096
	(-13.8, 3.0)		(-17.0, -0.1)	
Baseline Share Above Guidelines				
Below median (0-35.7%)	-1.8	0.644	-0.8	0.644
	(-5.3, 1.7)		(-4.1, 2.5)	
Above median (>357%)	-10.6	0.005	-10.9	0.005
	(-18.0, -3.2)		(-18.3, -3.6)	
Difference in effect	-8.8	0.034	-10.2	0.027
	(-17.0, -0.6)		(-18.2, -2.1)	

Notes: Differences in effect sizes were evaluated for statistical significance by scaling the difference in individual estimates above and below the median, scaled by the pooled standard error of the two differences. P-values are adjusted for multiple testing using a false discovery rate of 0.05. Difference in effect of the guideline intervention above and below the median procedure volume was significant prior to the FDR multiple testing adjustment.

© 2024 Wagner Z, et al. *JAMA Health Forum.*2024. All rights reserved.

eTable 6. Effect of social norms-based nudges on primary and secondary outcomes (discharge level)

	Effect of Peer Comparison Intervention (Percentage Points)				Effect of Guidelines Intervention (Percentage Points)			
	Unadjusted (95% CI)	p-value	Adjusted (95% CI)	p-value	Unadjusted (95% CI)	p-value	Adjusted (95% CI)	p-value
Primary Outcome								
Opioid prescriptions above guidelines (%)	-9.3 (-20.5, 1.9)	0.109	-9.7 (-14.5, -4.9)	0.000	-11.4 (-22.2, -0.7)	0.084	-10.1 (-15.6, -4.7)	0.001
Secondary Outcomes								
Morphine milligram equivalent (mean)	-9.3 (-32.4, 13.8)	0.530	-12.4 (-24.2, -0.6)	0.071	10.9 (-23.0, 44.8)	0.530	-12.2 (-25.2, 0.8)	0.071
Any opioid prescribed (%)	-4.6 (-16.7, 7.5)	0.554	-4.9 (-8.8, -1.1)	0.022	-3.6 (-15.6, 8.3)	0.554	-4.9 (-9.0, -0.8)	0.022

Unadjusted models compare means between each intervention arm and the control arm with standard errors clustered by specialty within hospital. Adjusted models control for baseline level of the outcome at the surgeon level. P-values are adjusted for multiple testing using a false discovery rate of 0.05.

© 2024 Wagner Z, et al. *JAMA Health Forum.*2024. All rights reserved.

eTable 7. Intervention effects on patient outcomes

Outcome	Effect of Peer Comparison Intervention HLM Estimate (95% CI)	Effect of Peer Comparison Intervention p-value	Effect of Guidelines Intervention HLM Estimate (95% CI)	Effect of Guidelines Intervention p-value
Any additional opioid refills within 30 days post-discharge	0.039 (-0.030, 0.108)	0.539	0.019 (-0.046, 0.084)	0.571
Any emergency department visits within 30 days post-discharge	-0.002 (-0.023, 0.192)	0.854	0.002 (-0.019, 0.024)	0.854
Any inpatient visits within 30 days post-discharge	0.006 (-0.001, 0.013)	0.157	0.001 (-0.005, 0.007)	0.671

Notes: Estimates are from hierarchical models that include random effects for surgeon and specialty within hospital. Additional opioid refills include only prescriptions made after the initial discharge. P-values are adjusted for multiple testing using a false discovery rate of 0.05.

© 2024 Wagner Z, et al. *JAMA Health Forum.*2024. All rights reserved.

eTable 8. Sensitivity analyses using surgeons who were more exposed to the intervention

	Restricting to surgeons who received and email (in the treatment groups) or met the criteria for receiving an email (in the control group)				Restricting to surgeons who prescribe opioids at discharge or discharged the patient themself			
	Effect of Peer Comparison Intervention		Effect of Guidelines Intervention		Effect of Peer Comparison Intervention		Effect of Guidelines Intervention	
	Adjusted (95% CI)	p-value	Adjusted (95% CI)	p-value	Adjusted (95% CI)	p-value	Unadjusted (95% CI)	p-value
Primary Outcome								
Opioid prescriptions above guidelines (%)	-7.8	<0.001	-9.3	<0.001	-4.5	0.057	-4.7	0.057
	(-12.0, -3.6)		(-13.4, -5.1)		(-9.2, 0.1)		(-9.2, -0.1)	
Secondary Outcomes								
Morphine milligram equivalent (mean)	-12.6	0.214	-7.3	0.314	-3.3	0.901	-0.9	0.901
	(-28.1, 2.9)		(-22.1, 7.4)		(-18.3, 11.8)		(-15.7, 13.9)	
Any opioid prescribed (%)	-3.0	0.299	-1.8	0.348	0.4	0.911	1.4	0.911
	(-7.1, 1.1)		(-5.4, 1.9)		(-6.1, 6.9)		(-4.9, 7.7)	

Adjusted models include random effects for surgeon and specialty within hospital and control for baseline level of the outcome at the surgeon level. P-values are adjusted for multiple testing using a false discovery rate of 0.05.

© 2024 Wagner Z, et al. *JAMA Health Forum.*2024. All rights reserved.

Supplementary Figures

eFigure 1. Example of Peer Comparison Intervention Email

Dear Dr. [name],

In an effort to reduce opioid use among our surgical patients, Sutter Health is reviewing opioid prescriptions and prescribing patterns for surgeons and will be communicating the findings.

In August, at least 2 of your patients were discharged with opioid prescriptions exceeding the amounts prescribed by 42% of your peers for these procedures.

42% of general surgeons at Sutter Health prescribe within the ranges below.

We will continue to send you opioid prescribing safety reports.

Sincerely,

[name]
Title
Hospital

Procedure	Amount prescribed by your peers (5mg oxycodone tablets)*
Colon or small bowel surgery	0-15
MIS cholecystectomy or appendectomy	0-8

*5mg oxycodone = 7.5mg hydrocodone = 75mg tramadol

For questions or to request information about how your prescribing is compared to your peers, please contact PostOp@sutterhealth.org. Do not reply to this email with any PHI or identifiable patient information.

This surgeon would have prescribed opioid quantitates over guideline-recommended amounts for two eligible procedures in August of 2022. The number 2 in red indicates the total number of eligible discharges in that month that included a guideline-discordant prescription.

© 2024 Wagner Z, et al. *JAMA Health Forum.*2024. All rights reserved.

eFigure 2. Example of Guidelines Intervention Email

Dear Dr. [name],

In an effort to reduce opioid use among our surgical patients, Sutter Health is reviewing opioid prescriptions and prescribing patterns for surgeons and will be communicating the findings.

In May, at least 2 of your patients were discharged with opioid prescriptions exceeding the amounts recommended by safety guidelines for these procedures.

For patient safety, Sutter Health recommends prescribing within the ranges below for these procedures. Doing so will also meet best practice safety guidelines for post-operative opioid prescribing.

We will continue to send you opioid prescribing safety reports.

Sincerely,

[name]
Title
Hospital name

Procedure	Amount recommended by Sutter Health (5mg oxycodone tablets)*
Colon or small bowel surgery	0-15
MIS cholecystectomy or appendectomy	0-8

* 5mg oxycodone = 7.5mg hydrocodone = 75mg tramadol

For questions or to request information about safety guidelines, please contact PostOp@sutterhealth.org. Do not reply to this email with any PHI or identifiable patient information.

This surgeon would have prescribed opioid quantitates over guideline recommended amounts for two eligible procedures in May of 2022. The number 2 in red indicates the total number of eligible discharges in that month that included a guideline-discordant prescription.

© 2024 Wagner Z, et al. *JAMA Health Forum.*2024. All rights reserved.

eFigure 3. Effect of social norms-based nudges on each quantile of MMEs prescribed at discharge

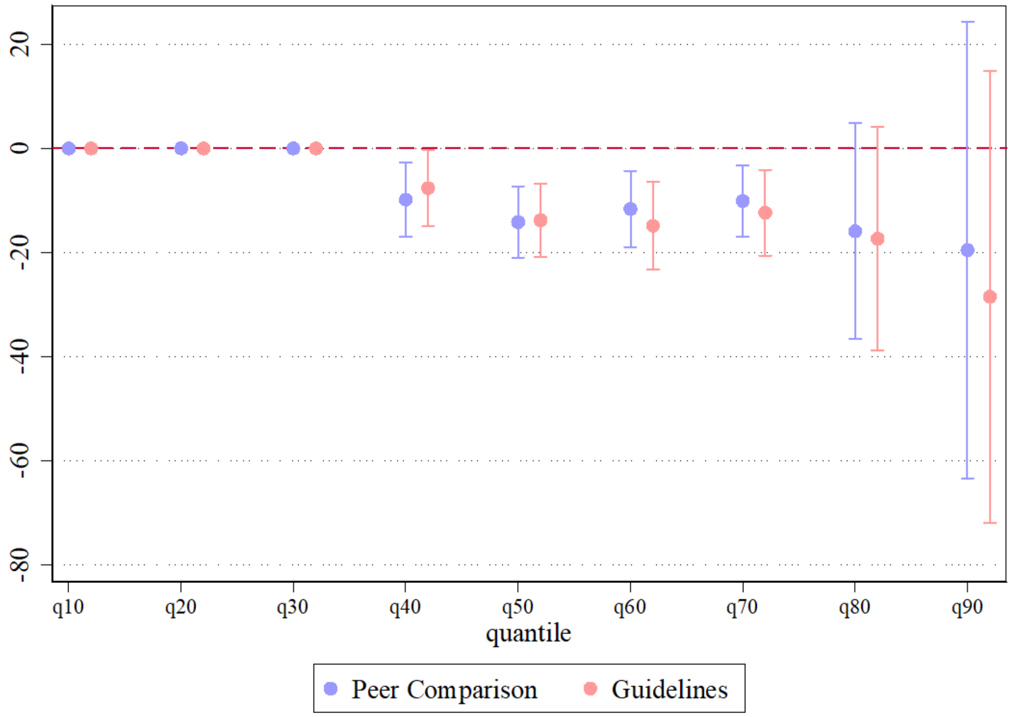

The intervention effects at each quantile were estimated using a separate quantile regression adjusted for baseline guideline-discordant prescribing and with standard errors cluster at the level of random assignment (specialty within hospital). Error bars are 95% confidence intervals. The 10th, 20th, and 30th percentile was zero for all three arms, which is why the coefficients are zero with no confidence interval.

© 2024 Wagner Z, et al. *JAMA Health Forum.*2024. All rights reserved.

eFigure 4. Exposure to interventions

A. Number of emails received by surgeons in intervention arms

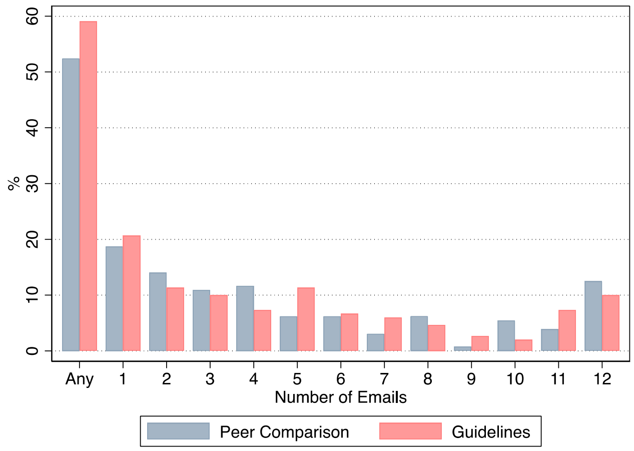

B. Share of patients that were discharge by surgeon who received an email in intervention arms

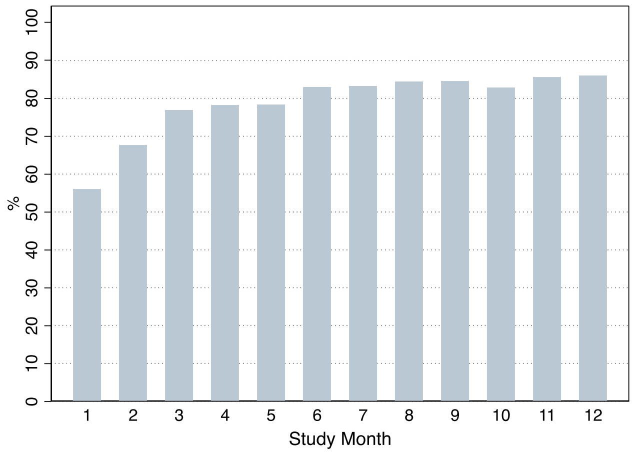

Panel A shows the share of surgeons who received each number of emails over the 12-month study period. "Any" indicates whether they received at least one email during the 12 months. Panel B shows the share of discharged patients in each month who had an operating surgeon who had previously received email feedback from either intervention arm.

© 2024 Wagner Z, et al. *JAMA Health Forum.*2024. All rights reserved.

Supplementary Appendix References

1. Raudenbush SW, Bryk AS. Hierarchical linear models: applications and data analysis methods. Thousand Oaks, CA: SAGE Publications, 2002.
2. Graubard B, Edward L, Korn E. Predictive margins with survey data. Biometrics 1999;55(2):652–659.
3. Kahan BC, Li F, Copas AJ, Harhay MO. Estimands in cluster-randomized trials: choosing analyses that answer the right question. Int J Epidemiol 2023;52(1):107-118.
4. Abadie A, Athey S, Imbens GW, Wooldridge J. When should you adjust standard errors for clustering? National Bureau of Economic Research, 2017.

© 2024 Wagner Z, et al. *JAMA Health Forum.*2024. All rights reserved.
