# Supplementary material for: Peer Comparison or Guideline-Based Feedback and Postsurgery Opioid Prescriptions: A Randomized Clinical Trial
Source: JAMA Health Forum. 2024 Mar 15;5(3):e240077. doi: 10.1001/jamahealthforum.2024.0077 (PMC10943416; doi:10.1001/jamahealthforum.2024.0077)
Supplement: Supplement 3. — Data Sharing Statement [file jamahealthforum-e240077-s003.pdf]

# Data Sharing Statement

Wagner. Peer Comparison or Guideline-Based Feedback and Postsurgery Opioid Prescriptions. *JAMA Health Forum*. Published March 15, 2024.
doi:10.1001/jamahealthforum.2024.0077

**Data**
**Data available:** Yes
**Data types:** Deidentified participant data, Data dictionary
**How to access data:** Deidentified data will be made available on reasonable request to the corresponding author (kwatkins@rand.org). Data aggregation of patents or providers may be used to prevent identifiability by inference.
**When available:** With publication

**Supporting Documents**
**Document types:** None

**Additional Information**
**Who can access the data:** researchers whose proposed use of the data has been approved
**Types of analyses:** for any purpose
**Mechanisms of data availability:** with a signed data access agreement
